# Dietary fruit and vegetable intake, gut microbiota, and type 2 diabetes: results from two large human cohort studies

**DOI:** 10.1186/s12916-020-01842-0

**Published:** 2020-12-03

**Authors:** Zengliang Jiang, Ting-yu Sun, Yan He, Wanglong Gou, Luo-shi-yuan Zuo, Yuanqing Fu, Zelei Miao, Menglei Shuai, Fengzhe Xu, Congmei Xiao, Yuhui Liang, Jiali Wang, Yisong Xu, Li-peng Jing, Wenhua Ling, Hongwei Zhou, Yu-ming Chen, Ju-Sheng Zheng

**Affiliations:** 1 Key Laboratory of Growth Regulation and Translational Research of Zhejiang Province, School of Life Sciences, Westlake University, Hangzhou, China; 2 Institute of Basic Medical Sciences, Westlake Institute for Advanced Study, Hangzhou, China; 3 Guangdong Provincial Key Laboratory of Food, Nutrition and Health; Department of Epidemiology, School of Public Health, Sun Yat-sen University, Guangzhou, China; 4 Microbiome Medicine Center, Division of Laboratory Medicine, Zhujiang Hospital, Southern Medical University, Guangzhou, China; 5 State Key Laboratory of Organ Failure Research, Southern Medical University, Guangzhou, China; 6 Department of Epidemiology, School of Public Health, Lanzhou University, Gansu, China; 7 Westlake Laboratory of Life Sciences and Biomedicine, Hangzhou, China

**Keywords:** Fruit and vegetable, Gut microbiota, Metabolites, Type 2 diabetes, Cohort

## Abstract

**Background:**

Little is known about the inter-relationship among fruit and vegetable intake, gut microbiota and metabolites, and type 2 diabetes (T2D) in human prospective cohort study. The aim of the present study was to investigate the prospective association of fruit and vegetable intake with human gut microbiota and to examine the relationship between fruit and vegetable-related gut microbiota and their related metabolites with type 2 diabetes (T2D) risk.

**Methods:**

This study included 1879 middle-age elderly Chinese adults from Guangzhou Nutrition and Health Study (GNHS). Baseline dietary information was collected using a validated food frequency questionnaire (2008–2013). Fecal samples were collected at follow-up (2015–2019) and analyzed for 16S rRNA sequencing and targeted fecal metabolomics. Blood samples were collected and analyzed for glucose, insulin, and glycated hemoglobin. We used multivariable linear regression and logistic regression models to investigate the prospective associations of fruit and vegetable intake with gut microbiota and the association of the identified gut microbiota (fruit/vegetable-microbiota index) and their related fecal metabolites with T2D risk, respectively. Replications were performed in an independent cohort involving 6626 participants.

**Results:**

In the GNHS, dietary fruit intake, but not vegetable, was prospectively associated with gut microbiota diversity and composition. The fruit-microbiota index (FMI, created from 31 identified microbial features) was positively associated with fruit intake (*p* < 0.001) and inversely associated with T2D risk (odds ratio (OR) 0.83, 95%CI 0.71–0.97). The FMI-fruit association (*p* = 0.003) and the FMI-T2D association (OR 0.90, 95%CI 0.84–0.97) were both successfully replicated in the independent cohort. The FMI-positive associated metabolite sebacic acid was inversely associated with T2D risk (OR 0.67, 95%CI 0.51–0.86). The FMI-negative associated metabolites cholic acid (OR 1.35, 95%CI 1.13–1.62), 3-dehydrocholic acid (OR 1.30, 95%CI 1.09–1.54), oleylcarnitine (OR 1.77, 95%CI 1.45–2.20), linoleylcarnitine (OR 1.66, 95%CI 1.37–2.05), palmitoylcarnitine (OR 1.62, 95%CI 1.33–2.02), and 2-hydroglutaric acid (OR 1.47, 95%CI 1.25–1.72) were positively associated with T2D risk.

**Conclusions:**

Higher fruit intake-associated gut microbiota and metabolic alteration were associated with a lower risk of T2D, supporting the public dietary recommendation of adopting high fruit intake for the T2D prevention.

## Background

Type 2 diabetes (T2D) prevalence is increasing rapidly throughout the world with an estimated global prevalence of 552 million by 2030 [1]. Fruits and vegetables are both essential components of a healthy dietary pattern [2], which are suggested to play an important role in T2D prevention. However, results from human prospective cohort studies have been inconsistent and the evidence linking fruit and vegetable intake and T2D is weak [3–5]. An alternative way to investigate the potential role of fruit and vegetable intake in T2D prevention is to examine the prospective association of fruit and vegetable intake with gut microbiota and explore its implication in the T2D development, given that gut microbiota is closely involved in the T2D etiology [6–9]. Yet, so far, little is known about whether and how habitual fruit and vegetable intake could influence gut microbiota structure and composition over a period of time. Evidence from large prospective human cohort studies is lacking [10, 11].

Mechanisms linking fruit and vegetable intake and T2D are mainly attributed to their rich sources of fibers, flavonoids, and various antioxidant compounds, which are reported to interact with the gut microbes and affect gut microbiota ecology [12, 13]. Therefore, we hypothesize that gut microbiota is a key mediator linking fruit and vegetable intake and T2D development. To date, only a limited number of cross-sectional cohort studies have shown an association between fruit and vegetable intake and gut microbiota composition [14–16]. Several other cross-sectional studies suggest that a dietary pattern which is rich in fruits and vegetables is associated with variations in gut microbiota composition [10, 17, 18]. Fecal metabolome analysis may provide novel evidence for the understanding of the relationship between fruit and vegetable intake, gut microbiota, and T2D, yet research in this area is sparse.

Therefore, this study aimed to investigate the prospective association of fruit and vegetable intake with the gut microbiota and to examine the association of fruit or vegetable-related gut microbiota and metabolites with T2D risk in a prospective cohort, including 1879 participants from the Guangzhou Nutrition and Health Study (GNHS) [19]. Replications of the above associations were subsequently conducted in an independent large cohort study including 6626 participants from the Guangdong Gut Microbiome Project (GGMP) [16].

## Methods

### Study design

This study was based on the GNHS, a community-based prospective cohort including 4048 participants of Han Chinese ethnicity [19]. Briefly, a total of 4048 participants, 40–75 years and living in Southern China Guangzhou City, were recruited into the GNHS between 2008 and 2013. Fecal samples of the participants were collected at one time point during a follow-up visit of the participants to the study site up to Apr 30, 2019 (median follow-up of 6.2 years from entry into the cohort). We excluded the participants who were (1) without valid questionnaire information on dietary intake (including fruit intake, and vegetable intake) at baseline (*n* = 47); (2) self-reported baseline cancers, chronic renal dysfunction, or cirrhosis (*n* = 24); (3) missing covariates (age, gender, BMI, education, income, smoke, alcohol status, total energy intake, and physical activity) (*n* = 2); (4) extreme levels of total energy intake (men, < 800 kcal or > 4000 kcal; women, < 500 kcal or > 3500 kcal) (*n* = 41); (5) without measurement of gut microbiota data during follow-up (*n* = 2038); and (6) with antibiotic use within 2 weeks (*n* = 17) of stool collection. Finally, 1879 participants were included in the present analysis. T2D was defined as fasting blood glucose ≥ 7.0 mmol/L or glycated hemoglobin (HbAlc) ≥ 6.5% or currently under medical treatment for T2D, according to the American Diabetes Association’s diagnostic criteria [20]. Participant was diagnosed as a T2D case if meeting the above T2D criteria at baseline or/and during follow-up.

The GGMP is a large community-based cross-sectional cohort conducted between 2015 and 2016 including 7009 participants with high-quality gut microbiome data. The GGMP participants were from 14 randomly selected districts or counties in Guangdong province, China. In face-to-face questionnaire interviews, the host metadata including sociodemographic features, disease status, lifestyle, and dietary information (via food frequency questionnaire, FFQ) were collected [16]. We excluded the participants who were (1) without valid questionnaire information on dietary intake (including fruit intake and vegetable intake) (*n* = 140); (2) missing covariates (age, gender, BMI, education, smoke, alcohol status, and Bristol stool score) (*n* = 243). Finally, we included 6626 participants (52.8 ± 14.7 y, 55.2% of women) from GGMP in our analysis as an independent validation cohort. Characteristics of the included participants in the GGMP are presented in Additional file 1: Table S1. Detailed information regarding host metadata and stool sample collection and 16S rRNA gene sequencing process for GGMP have been reported previously [16].

### Measurement of dietary intakes and other covariates in GNHS

In GNHS, during the on-site face-to-face interviews, we collected information on socio-demographic, lifestyle, and dietary factors and medical history. Habitual dietary intakes over the past 12 months were assessed at baseline by a validated FFQ with 79-food items, as previously described [21]. The energy-adjusted correlation coefficients between the FFQ and 3-day diet records ranged from 0.30 to 0.68 for different food groups (for example, it was 0.37 for vegetable, 0.56 for fruit, and 0.48 for dairy products) [21]. The food items were grouped into the following groups: cereals (12 food items), beans, soy and nut (10 items), vegetables (13 items), fruits (10 items), animal-based foods (red meat, poultry, fish, eggs, and dairy products: 26 items), and drinks (8 items) [21]. Total energy intake was calculated according to the Chinese Food consumption Table, 2002 [22]. All food items were adjusted for total energy intake using the residual method [23]. The detailed items in the fruit and vegetable groups were provided in Additional file 1: Table S2. During the interview, all the participants were asked about the frequency of each fruit and vegetable they consumed and the average amount they consumed (50 g or 1 Liang was used as a common unit). Physical activity was assessed as total metabolic equivalent for task (MET) hours per day on the basis of a questionnaire for physical activity [24]. Anthropometric parameters, including weight, height, waist, and hip circumference, were measured by trained nurses at the site during the baseline interview. Fasting venous blood samples were taken at recruitment and follow-up visit and were aliquoted and stored in a − 80 °C freezer prior to analysis. Fasting glucose and insulin were measured by colorimetric methods using a Roche cobas 8000 c702 automated analyzer (Roche Diagnostics GmbH, Shanghai, China). High-performance liquid chromatography was used to measure HbAlc using the Bole D-10 Hemoglobin A1c Program on a Bole D-10 Hemoglobin Testing System. Homeostasis model assessment of insulin resistance (HOMA-IR) and β-cell function (HOMA-β) were calculated based on fasting glucose and insulin levels [25].

### Fecal sample collection, DNA extraction, and 16S rRNA gene sequencing in GNHS

During a follow-up visit to the study center, participants were given a stool sampler and provided detailed instructions for the stool sample collection. Briefly, each participant collected their stool sample after defecation, recorded its Bristol stool score in the stool sampler, and gave the sample to the staff immediately. The stool samples with ice bag were transported to the research laboratory and stored in a − 80 °C freezer within 4 h. Stool samples that were not delivered to the collection point within 4 h were discarded. Detailed information regarding DNA extraction, gut microbiota 16S rRNA gene sequencing, and fecal metabolic profiles in GNHS is provided in Additional file 1: Method S1 and Method S2 [26–28].

### Targeted fecal metabolomics profiling in GNHS

The targeted metabolomics profiling of fecal samples (*n* = 1017) was performed by Metabo-Profile (Shanghai, China). Detailed information regarding targeted fecal metabolomics profiling in GNHS is provided in Additional file 1: Method S3.

### Statistical analysis

We examined participant characteristics using proportions and mean values with corresponding SDs. We categorized fruit and vegetable intake in quartiles with the lowest quartile indicating low intake.

In GNHS, we examined the associations of baseline fruit and vegetable intakes with *α*-diversity indices (Observed species, Shannon index and Chao 1 index) using a multivariable linear regression model, adjusted for Bristol stool score, sequencing run, sequencing depth, age, sex, BMI, smoking status, alcohol status, physical activity, education, income, T2D status, drug use (medications for hypertension, hyperlipidemia and T2D), total energy intake, dietary intake of vegetable /fruit (mutual adjustment for each other), red and processed meat, fish, and dairy products [6, 10, 11, 29–31]. The association between fruit and vegetable intakes and β-diversity dissimilarity based on Bray-Curtis distance was tested using permutational ANOVA (PERMANOVA) (999 permutations) [3], adjusted for the same covariates as above analyses of *α*-diversity indices.

We used Multivariate Analysis by Linear Models (MaAsLin) to identify potential gut microbial operational taxonomic units (OTUs) associated with dietary fruit or vegetable intake, adjusted for the same covariates as above diversity analysis. The Benjamini-Hochberg method was used to control false discovery rate (FDR) due to multiple testing.

To summarize the association of fruit and vegetable with the gut microbes, we calculated a fruit-microbiota index (FMI), vegetable-microbiota index (VMI), and total fruit and vegetable-microbiota index (TFVMI) based on the identified OTUs for each of the three dietary variables (Additional file 1: Method S4).

To test the validity of the above created microbiota index, we used a linear regression model to examine the association of fruit, vegetable, or their sum with the corresponding microbiota index, adjusted for the same covariates as the above fruit/vegetable-microbiota analysis. To further test the robustness of the associations and minimize the influence of disease status, we repeated the analysis in non-T2D participants using the linear regression models, adjusted for the same covariates. To gain insight about the relationship between the different fruit types and FMI, we used the partial correlation analysis to investigate the correlation of FMI with different fruit types, adjusted for age, sex, and BMI.

We then used a multivariable logistic regression model to examine the cross-sectional association of FMI, VMI, or TFVMI with T2D risk in the GNHS, adjusted for Bristol stool score, sequencing run, sequencing depth, age, sex, BMI, smoking status, alcohol status, physical activity, education, income, drug use (medications for hypertension, hyperlipidemia), total energy intake, dietary intake of vegetable /fruit (mutual adjustment for each other), red and processed meat, fish, and dairy products. We also used a multivariable linear regression model to examine the association of the fruit/vegetable-microbiota index with T2D-related traits (fasting serum insulin, glucose, HbAlc, HOMA-IR, and HOMA-β), adjusted for the same covariates as above fruit/vegetable-microbiota analysis.

In the GGMP participants, we created the same FMI, VMI, or TFVMI using the above identified OTUs to replicate the results from the GNHS. We used a multivariable linear regression to examine the association of corresponding dietary factor with the related microbiota index, adjusting for Bristol stool score, age, sex, BMI, smoking status, alcohol status, education, T2D status, dietary intake of vegetable/fruit (mutual adjustment for each other), and red and processed meat. The analyses were conducted among all GGMP participants and among those without T2D, respectively. We also used a logistic regression to examine the association of the FMI, VMI, or TFVMI with T2D risk, adjusted for Bristol stool score, age, sex, BMI, smoking status, alcohol status, education, dietary intake of vegetable /fruit (mutual adjustment for each other), and red and processed meat. For GGMP, we did not include the income in the statistical models due to large amount of missing values (income data were available among 4109 out of 6626 participants). We therefore did a sensitivity analysis with further adjustment for income in the above analyses to examine the robustness of the models. Then, for each of the above linear regression or logistic regression, the effect estimates from GNHS and GGMP were pooled by random effects meta-analysis.

To gain further mechanistic insight about the connection between fruit and vegetable intake and T2D risk, we investigated the correlation of the FMI, VMI, or TFVMI with fecal metabolome with partial correlation analysis in the GNHS, adjusted for age, sex, and BMI. We further examined the association of the above identified fecal metabolites with T2D risk using logistic regression, adjusted for the same covariates as the above FMI/VMI/TFVMI-T2D analysis. Throughout the above analyses, FDR from multiple testing was controlled by the Benjamini-Hochberg method.

We used the co-occurrence network analysis based on the above partial correlation coefficient to demonstrate the interaction of the above gut microbial OTUs and metabolites respectively, and only the significant correlations (larger than 0.3 or smaller than − 0.3) were used for network construction. The networks were further visualized in Cytoscape software version 3.7.2. Pathways enrichment analysis of metabolomics profiles was performed by MetaboAnalyst 4.0 [32] using the online server. We used R version 3.6.3 for statistical analysis unless otherwise specified, and *p* value < 0.05 was considered statistically significant.

## Results

### Characteristics of study participants

In GNHS, the mean (SD) age was 58.6 (6.1) years, with 67.3% women participants (Table 1). At baseline, the mean (SD) intake of fruit, vegetable, and their sum were 146 (109), 383 (182), and 529 (239) g/day, respectively (Additional file 1: Table S3). The fruit, vegetable, and total fruit and vegetable intakes were significantly different between men and women (*p* < 0.001, *p* = 0.025, *p* < 0.001, respectively) (Additional file 1: Table S4). For GGMP, the mean (SD) age was 52.8 (14.7) years, with 55.2% women participants. The mean (SD) intake of fruit, vegetable, and their sum were 79 (117), 337 (230), and 416 (272) g/day, respectively (Additional file 1: Table S1).

**Table 1:** Characteristics of the study participants in the Guangzhou Nutrition and Health Study

Characteristics	Total	Fruit intake					Vegetable intake				
		Q1	Q2	Q3	Q4	*p-*trend	Q1	Q2	Q3	Q4	*p*-trend
*n*	1879	471	471	467	470		470	470	469	470	
Age, years	58.6 (6.1)	59.1 (6.5)	58.8 (6.4)	58.5 (6.0)	58.0 (5.5)	0.005	58.8 (6.9)	59.0 (6.2)	58.4 (5.7)	58.3 (5.4)	0.079
Sex, *n* (% of women)	1264 (67.3)	278 (59.0)	297 (63.1)	335 (71.7)	354 (75.3)	< 0.001	291 (61.9)	305 (64.9)	332 (70.8)	336 (71.5)	< 0.001
BMI, kg/m^2^	23.2 (3.0)	23.3 (3.1)	23.4 (3.2)	23.1 (2.9)	23.3 (2.8)	0.683	23.3 (3.1)	23.4 (3.0)	23.0 (2.7)	23.3 (3.1)	0.420
Total energy intake, kcal/day	1742 (488)	1573 (469)	1687 (455)	1765 (432)	1944 (517)	< 0.001	1505 (433)	1677 (430)	1798 (455)	1989 (500)	< 0.001
Physical activity, MET hours/day	40.6 (14.1)	38.5 (13.4)	39.6 (13.6)	41.8 (14.3)	42.6 (14.6)	< 0.001	38.8 (12.7)	39.5 (13.6)	41.6 (14.3)	42.5 (15.2)	< 0.001
Vegetable intake, g/day	383 (182)	317 (175)	352 (149)	401 (157)	464 (208)	< 0.001	192 (45)	303 (29)	414 (37)	624 (162)	< 0.001
Fruit intake, g/day	146 (109)	42 (19)	95 (16)	154 (19)	292 (107)	< 0.001	103 (86)	133 (92)	154 (97)	194 (133)	< 0.001
Total fruit and vegetable intake, g/day	529 (239)	358 (178)	448 (152)	556 (159)	756 (248)	< 0.001	295 (100)	436 (100)	568 (105)	819 (222)	< 0.001
Red and processed meat intake, g/day	104 (61)	98 (59)	101 (56)	105 (57)	114 (70)	< 0.001	89 (54)	99 (54)	112 (64)	117 (67)	< 0.001
Fish intake, g/day	50 (51)	44 (64)	43 (31)	54 (60)	61 (42)	< 0.001	37 (56)	45 (34)	53 (44)	67 (62)	< 0.001
Dairy products intake, g/day	115 (114)	88 (104)	107 (108)	124 (122)	142 (116)	< 0.001	94 (105)	113 (107)	125 (115)	128 (127)	< 0.001
Current alcohol drinker, *n* (%)	137 (7.3)	33 (7.0)	51 (10.8)	28 (6.0)	25 (5.3)	0.065	39 (8.3)	40 (8.5)	32 (6.8)	26 (5.5)	0.063
Current smoker, *n* (%)	292 (15.5)	100 (21.2)	87 (18.5)	57 (12.2)	48 (10.2)	< 0.001	93 (19.8)	79 (16.8)	59 (12.6)	61 (13.0)	0.001
Income level, *n* (%)						< 0.001					0.472
≤ 500 ¥/months	28 (1.5)	11 (2.3)	4 (0.8)	8 (1.7)	5 (1.1)		6 (1.3)	8 (1.7)	9 (1.9)	5 (1.1)	
501–1500 ¥/months	403 (21.4)	116 (24.6)	110 (23.4)	93 (19.9)	84 (17.9)		85 (18.1)	98 (20.9)	105 (22.4)	115 (24.4)	
1501–3000 ¥/months	1197 (63.7)	301 (63.9)	299 (63.5)	295 (63.2)	302 (64.2)		343 (73.0)	302 (64.2)	279 (59.5)	273 (58.1)	
> 3000 ¥/months	251 (13.4)	43 (9.1)	58 (12.3)	71 (15.2)	79 (16.8)		36 (7.6)	62 (13.2)	76 (16.2)	77 (16.4)	
Education, *n* (%)						0.492					0.130
Middle school or lower	510 (27.1)	139 (29.5)	120 (25.5)	128 (27.4)	123 (26.2)		132 (28.1)	137 (29.1)	111 (23.7)	130 (27.7)	
High school or professional college	864 (46.0)	205 (43.5)	201 (42.7)	218 (46.7)	240 (51.1)		203 (43.2)	217 (46.2)	226 (48.2)	218 (46.3)	
University	505 (26.9)	127 (27.0)	150 (31.8)	121 (25.9)	107 (22.7)		135 (28.7)	116 (24.7)	132 (28.1)	122 (26.0)	
Glucose, mmol/L	5.48 (1.32)	5.51 (1.22)	5.53 (1.65)	5.54 (1.38)	5.34 7(0.87)	0.089	5.49 (1.24)	5.39 (1.05)	5.57 (1.64)	5.47 (1.27)	0.657
Insulin, μU/mL	7.30 (4.00)	7.17 (4.00)	7.42 (4.00)	7.41 (4.43)	7.19 (3.50)	0.926	7.54 (4.36)	7.41 (4.05)	7.12 (3.64)	7.09 (3.87)	0.072
HbAlc, %	7.24 (4.22)	7.13 (4.01)	7.26 (4.25)	7.14 (4.03)	7.42 (4.57)	0.396	7.13 (3.95)	7.28 (4.64)	7.37 (4.51)	7.16 (3.70)	0.822
HOMA-IR	1.83 (1.23)	1.81 (1.25)	1.86 (1.16)	1.91 (1.50)	1.74 (0.91)	0.626	1.89 (1.33)	1.81 (1.19)	1.81 (1.10)	1.81 (1.29)	0.356
HOMA-β, %	85.2 (54.2)	83.8 (54.5)	87.6 (58.2)	83.8 (56.1)	85.7 (47.3)	0.873	88.5 (61.5)	88.7 (52.8)	81.3 (50.8)	81.5 (49.9)	0.023
Medication use, *n* (%)						0.057					0.930
Hypertension	100 (5.3)	22 (4.7)	26 (5.5)	26 (5.6)	26 (5.5)		15 (3.2)	23 (4.9)	26 (5.5)	36 (7.7)	
Hyperlipidemia	111 (5.9)	31 (6.6)	39 (8.3)	18 (3.9)	23 (4.9)		35 (7.4)	25 (5.3)	31 (6.6)	20 (4.3)	
T2D	58 (3.1)	21 (4.5)	19 (4.0)	6 (1.3)	12 (2.6)		19 (4.0)	12 (2.6)	17 (3.6)	10 (2.1)	

Data are expressed as mean (SD) for continuous variables and *n* (%) for categorical variables; Q1 indicates the quartile with the lowest intake; *p*-trend represents the comparison among quartiles using linear regression

*Q1* quartile 1, *Q2* quartile 2, *Q3* quartile 3, *Q4* quartile 4, *HbAlc* glycated hemoglobin, *HOMA-IR* homeostasis model assessment of insulin resistance, *HOMA-β* homeostasis model assessment of β-cell function, *T2D* type 2 diabetes

### Prospective association of fruit and vegetable intake with gut microbiota

**Fig. 1:**
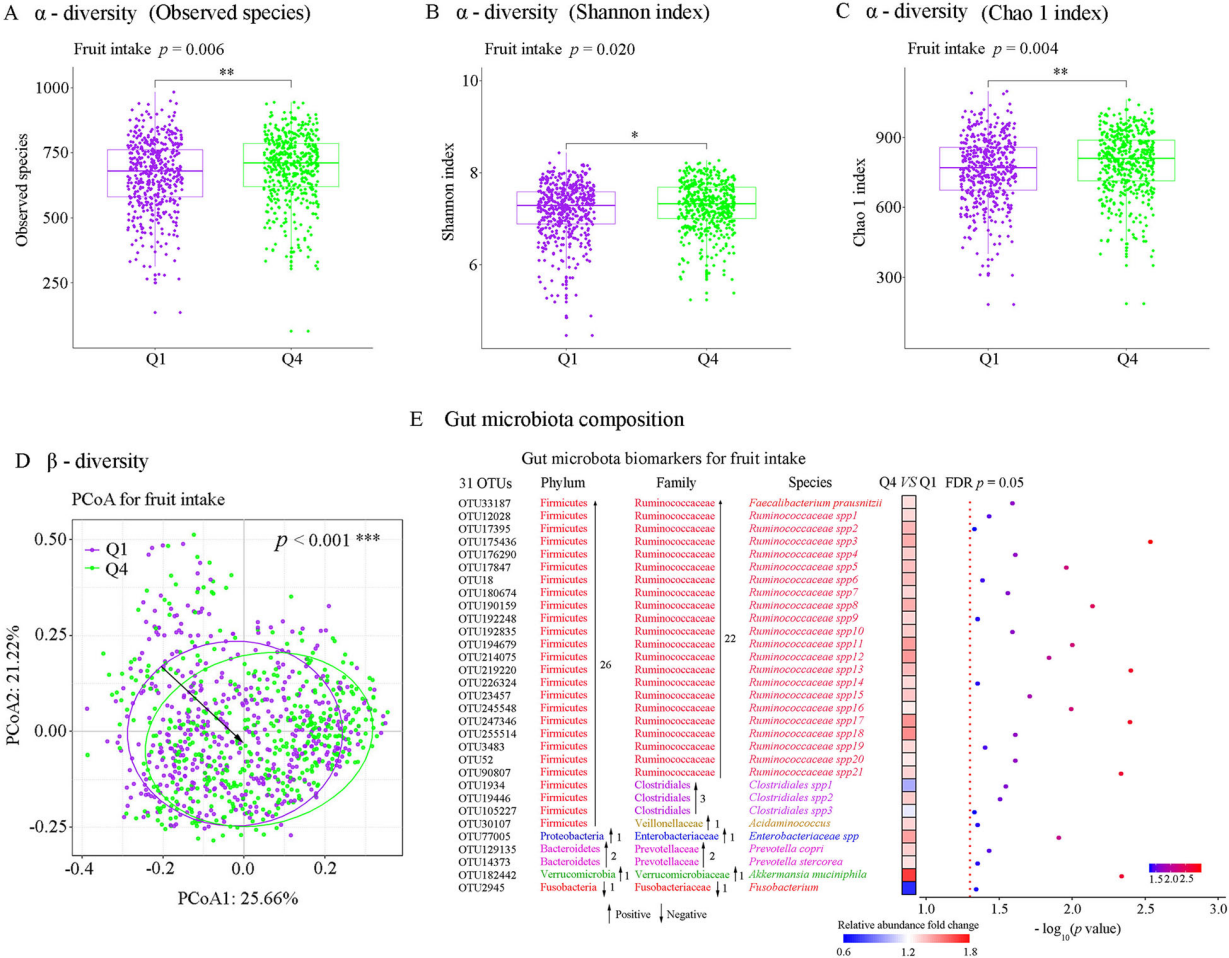
The prospective association of fruit intake with the overall human gut microbiota in the Guangzhou Nutrition and Health Study. **a**–**c** Results of different α-diversity matrix. **a** Observed species. **b** Chao 1’s diversity parameter. **c** Shannon’s diversity parameter. Multivariable linear regression was used to estimate the difference in α-diversity comparing extreme quartiles (quartile 4 versus quartile 1) of fruit intake, adjusted for Bristol stool score, sequencing run, sequencing depth, age, sex, BMI, physical activity, education, income, smoking status, alcohol status, drug use (medications for hypertension, hyperlipidemia and T2D), T2D status, total energy intake, dietary intakes of vegetable, red and processed meat, fish and dairy products. **d** β-diversity: principal coordinate analysis (PCoA) plot based on Bray-Cutis distance at operational taxonomic unit (OTU) level. Permutational ANOVA (PERMANOVA) (999 permutations) was used to identify the variation of β-diversity in human gut microbiota structure comparing extreme quartiles of fruit intake, adjusted for the same covariates. **e** MaAsLin was used to identify the gut microbial biomarkers for fruit intake comparing extreme quartiles of fruit intake, adjusted for the same covariates. The Benjamini-Hochberg method was used to adjust *p* values for multiple testing. Value with asterisk is significantly different (*p < 0.05, ** *p* < 0.01, ***p < 0.001)

In GNHS, habitual fruit intake was positively associated with Observed species (Q4 vs Q1: *p* = 0.006), Shannon index (Q4 vs Q1: *p* = 0.020), and Chao 1 index (Q4 vs Q1: *p* = 0.004) (Fig. 1a–c). Vegetable intake or total fruit and vegetable intake was not associated with any of the above α-diversity indices (Additional file 1: Figure S1). Fruit intake was significantly associated with the shift of β-diversity (*p* < 0.001) (Fig. 1d). However, associations of vegetable intake and total fruit and vegetable intake with β-diversity were not significant (Additional file 1: Figure S2).

Comparing the highest with lowest quartile, fruit intake was prospectively associated with 31 gut microbial OTUs. The identified 31 OTUs for fruit intake were assigned to *Faecalibacterium prausnitzii*, *Akkermansia muciniphila*, *Ruminococcaceae*, *Clostridiales*, *Acidaminococcus*, *Prevotella stercorea*, *Prevotella copri*, *Fusobacterium*, and *Enterobacteriaceae* (Fig. 1e). Thirty of the identified 31 OTU biomarkers were positively associated with fruit intake, whereas OTU2945_ *Fusobacterium* was negatively associated with fruit intake (Fig. 1e and Additional file 1: Table S5). Vegetable intake was only associated with 1 OTU belonging to *Lachnospira*, and total fruit and vegetable intake was associated with 2 OTUs belonging to *Lachnospira* and *Lachnospiraceae* spp. (Additional file 1: Table S6 and Table S7).

### Association of the fruit or vegetable-associated gut microbiota alteration with T2D

**Fig. 2:**
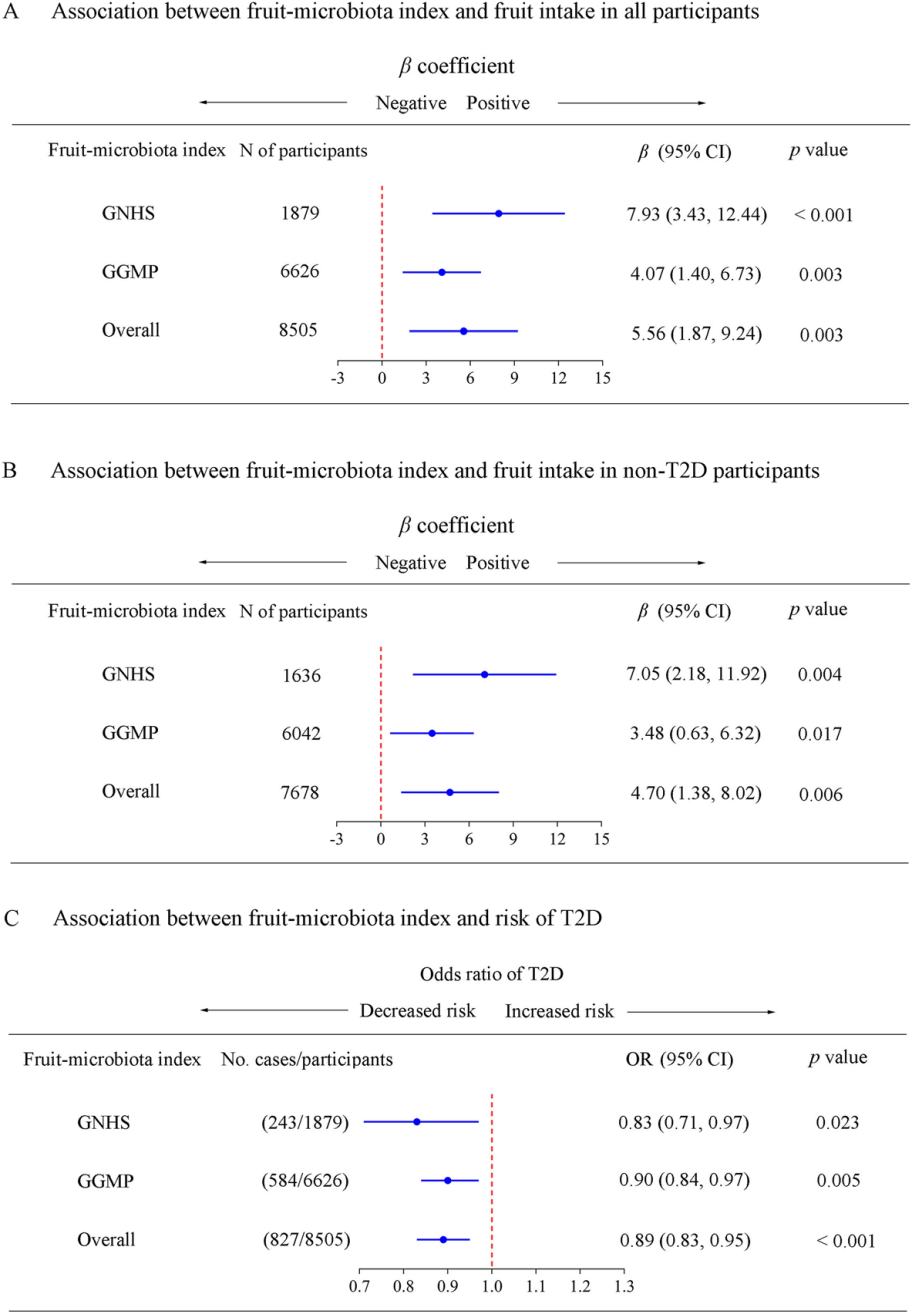
Relationships among the fruit intake, fruit-gut microbiota index, and type 2 diabetes. **a** Multivariable linear regression was used to estimate the associations of fruit intake with fruit-microbiota index (FMI) in all participants in the Guangzhou Nutrition and Health Study (GNHS), and the Guangdong Gut Microbiome Project (GGMP). **b** Multivariable linear regression was used to estimate the associations of fruit intake with FMI in non-T2D participants in the GNHS and GGMP. **c** Multivariable logistic regression was used to estimate the association of FMI (per standardized unit increase) with type 2 diabetes (T2D) risk in the GNHS and GGMP respectively. The effect estimates from GNHS and GGMP were pooled using random effects meta-analysis for each of the above analyses

In GNHS, fruit intake was positively associated with FMI among all the participants, as well as the non-T2D participants (*p* < 0.001 and *p* = 0.004, respectively) (Fig. 2a and B). FMI was positively correlated with dietary intake of mango, banana, apple, grape, and durian (Additional file 1: Figure S3). We found that per unit increment in FMI was associated with 17% lower risk of T2D (OR 0.83, 95%CI 0.71–0.97) (Fig. 2c). FMI was inversely associated with HbAlc (*p* = 0.013), and positively associated with HOMA-β (*p* = 0.038) (Additional file 1: Figure S4). However, VMI or TFVMI was not associated with corresponding dietary intake (Additional file 1: Figure S5A) or T2D (Additional file 1: Figure S5B).

**Fig. 3:**
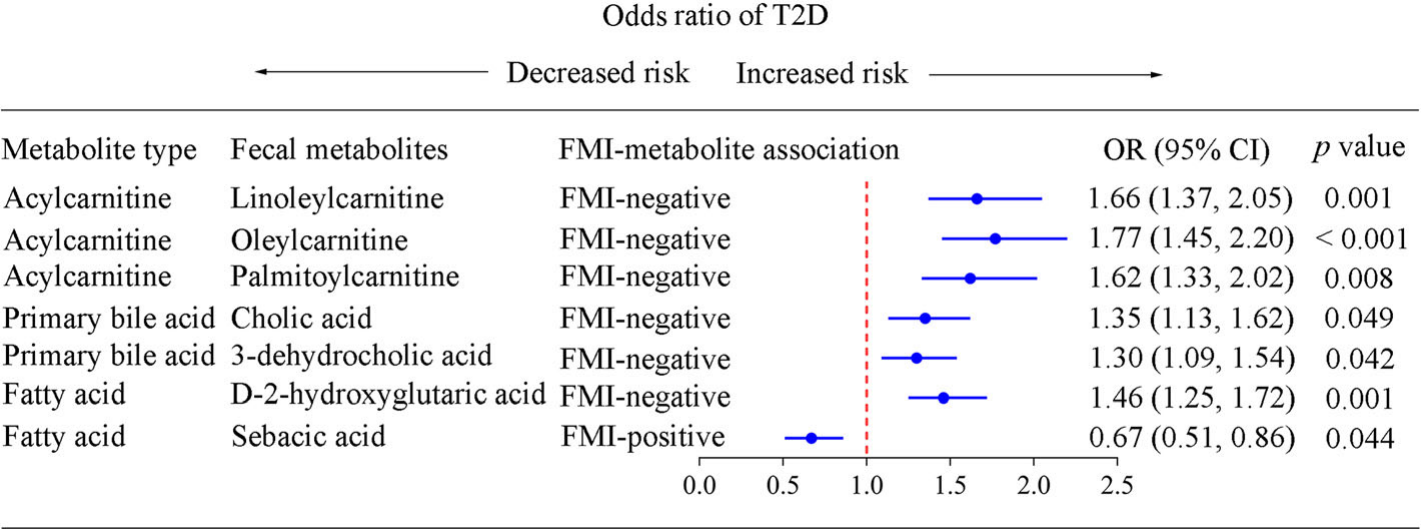
Association of the fruit-microbiota index-related fecal metabolites and type 2 diabetes. Multivariable logistic regression was used to examine the association of the fruit-microbiota index (FMI)-related fecal metabolites (per standardized unit increase) with type 2 diabetes (T2D) risk in the Guangzhou Nutrition and Health Study (133 cases/1017 participants), adjusted for Bristol stool score, sequencing run, sequencing depth, age, sex, BMI, physical activity, education, income, smoking status, alcohol status, drug use (medications for hypertension, hyperlipidemia, and T2D), total energy intake, dietary intakes of vegetable, red and processed meat, fish, and dairy products. “FMI-positive” and “FMI-negative” represented that fecal metabolites had positive and negative association with FMI, respectively. The Benjamini-Hochberg method was used to control the false discovery rate due to multiple testing. Adjusted *p* value < 0.05 is significantly different

In the GGMP, the FMI was significantly positively associated with fruit intake in all participants and non-T2D participants (*p* = 0.003 and *p* = 0.017, respectively) (Fig. 2a and B). Per unit increment in FMI was associated with 10% lower risk of T2D (OR: 0.90, 95%CI: 0.84–0.97) (Fig. 3c). In addition, results of the sensitivity analysis suggested that with and without including income as a covariate did not substantially affect the results (Additional file 1: Table S8). Meta-analysis of results from the two cohorts consistently showed that the FMI was significantly positively associated with fruit intake in all participants and non-T2D participants (*p* = 0.003 and *p* = 0.006, respectively) (Fig. 2a and b). Meta-analysis also suggested that per unit increment in FMI was associated with 11% lower risk of T2D (pooled OR 0.89, 95%CI 0.83–0.95) (Fig. 2c).

### Association of the FMI-associated fecal metabolites with T2D

In the GNHS, the FMI was significantly associated with 76 fecal metabolites which could be clustered into three modules (Additional file 1: Figure S6 and Figure S7). Pathway enrichment analysis of the identified metabolites showed that the FMI-related metabolites were mainly assigned to pathways of bile acid biosynthesis, fatty acid biosynthesis, and fatty acid metabolism (Additional file 1: Figure S8). Notably, 7 out of the 76 FMI-related metabolites were significantly associated with T2D risk (Fig. 3 and Additional file 1: Figure S7C). The FMI-positive related metabolite sebacic acid was inversely associated with T2D (OR 0.67, 95%CI 0.51–0.86), whereas the FMI-negative related metabolites cholic acid (OR 1.35, 95%CI 1.13–1.62), 3-dehydrocholic acid (OR 1.30, 95%CI 1.09–1.54), oleylcarnitine (OR 1.77, 95%CI 1.45–2.20), linoleylcarnitine (OR 1.66, 95%CI 1.37–2.05), palmitoylcarnitine (OR 1.62, 95%CI 1.33–2.02), and 2-hydroglutaric acid (OR 1.47, 95%CI 1.25–1.72) were positively associated with T2D (Fig. 3).

## Discussion

In the present large-scale epidemiological study, we demonstrated that fruit intake was prospectively associated with α-diversity, β-diversity, and 31 OTUs of gut microbiota, whereas the influence of vegetable intake on gut microbiota was minimal. The novel created FMI, which represented the microbial biomarker of fruit intake, was positively associated with fruit intake and inversely associated with T2D risk. We successfully replicated the FMI-fruit intake association and the FMI-T2D association in a large independent cohort study. Fecal metabolome analysis revealed specific fecal metabolites linking fruit-associated gut microbiota and T2D.

Fruit is an essential component of a healthy dietary pattern, which is suggested to play an important role in maintaining the balance of gut microbiota and improving intestinal ecology [17]. However, to date, only a limited number of cross-sectional cohort studies have shown an association between fruit intake and gut microbiota composition and little known about the prospective association of fruit intake with gut microbiota, and its implication for T2D [15–17]. Results from large prospective studies are important for the causal inference given that it is difficult and not feasible to conduct long-term large-scale randomized controlled trials for fruit and vegetable intake. Specifically, high fruit intake had positive association with 27 OTUs (out of total 31 OUTs) belonging to *Faecalibacterium prausnitzii*, *Akkermansia muciniphila*, *Ruminococcaceae*, *Clostridiales*, and *Acidaminococcus*, which indicated that high fruit intake was potentially beneficial for human health through increasing production of short-chain fatty acids, maintaining intestinal mucosal integrity, improving insulin sensitivity and anti-inflammatory properties [33–36]. In addition, high fruit intake was inversely associated with *Fusobacterium*, which was positively associated with T2D, ulcerative colitis, and colorectal cancer in prior studies [37–39].

As indicated in previous studies [6, 9], human gut microbiota plays a crucial role in the development of T2D. Given the weak evidence on the protective association of fruit and vegetable intake with T2D based on the self-reported questionnaire data [3, 4], identification of novel gut microbial biomarkers of fruit or vegetable intake may help clarify the relationship of fruit and vegetable intake with T2D risk. The present study demonstrated that the novel fruit microbiota index, which represented the microbial features of fruit intake, was positively associated with fruit intake and inversely associated with T2D risk. These findings collectively suggest that habitual fruit intake has the potential to reshape the human gut microbiome in a direction beneficial for the prevention of T2D. We did not find many vegetable-related gut microbiota, which may be because that majority of the vegetables consumed in Chinese cultures are deeply cooked, and therefore, the influence on gut microbiota is compromised. Therefore, in future work, it may be important to investigate the potential different associations with gut microbiota for raw versus cooked vegetables. In addition, impact of different fruit subgroups on the gut microbiota is also an interesting topic for further research.

Our data demonstrated that specific gut microbiota related metabolites contributed to the interpretation of the connection between the fruit-related microbiota and T2D. Previous studies demonstrated that treatment with specific microbiota derived secondary bile acids (obeticholic acid, DCA, and GDCA) in patients with T2D improved insulin sensitivity and HbAlc, which was consistent with our present study [40, 41]. Another study found that fecal sebacic acid was decreased in IBD patients [42]. High plasma levels of palmitoylcarnitine and linoleylcarnitine reflecting dysfunctional glucose and fatty acid metabolism were correlated with T2D, obesity, and cardiovascular disease [43, 44]. Taken together, our results suggest that higher fruit intake-related gut microbiota alteration may be beneficial for T2D prevention.

### Strengths and limitations

The present study had several strengths. First, it was based on a large prospective study, as the prospective relationship between fruit and vegetable intake and gut microbiota was rarely investigated in prior studies [15–18], which mainly focused on cross-sectional associations. Second, we constructed a novel gut microbial index for fruit intake and used it to demonstrate the potential beneficial association of fruit intake for T2D prevention. Third, we replicated our main findings in another large cohort study. Fourth, we identified several potential microbial metabolites linking the association between fruit-related gut microbiota and T2D.

The present study also contains several limitations. First, the dietary assessment is based on FFQ, which is subject to recall bias and measurement error. In addition, we could not obtain the information of cooking methods and intake of probiotic containing foods from FFQ and we did not measure the serum biomarkers of fruit and vegetable intake (i.e., different micronutrients). Nevertheless, FFQ is a commonly used tool in large-scale cohort study and it is suitable for ranking individuals within a cohort [45]. Second, diet was only assessed at one timepoint at baseline and it may change over time. Third, although we included fecal metabolites as objective biomarkers in our analysis, we did not measure some specific blood gut microbiota-related metabolites (such as lipopolysaccharides and Trimethylamine N-oxide), which may potentially help further improve the interpretation of our present findings. Fourth, the replication cohort (GGMP) is a cross-sectional study, while this is the best data resource and largest study we could find at current stage. Finally, our two cohorts are both based on individuals of Chinese ethnicity, which may not be generalizable to other populations or ethnicities.

## Conclusions

Results of the present study suggest that high fruit intake is prospectively associated with human gut microbiome, favoring the beneficial association of fruit intake with the T2D risk. Our study supports the emerging concept that healthy diet-shaped gut microbiota contributes to a decreased risk of T2D and other metabolic diseases. Meanwhile, our results provide important and timely evidence supporting the public dietary recommendation of adopting a healthy dietary pattern with high fruit intake for the T2D prevention.

## Supplementary information

**Additional file 1 : Method S1**. Fecal microbial DNA extraction and 16S rRNA gene sequencing in GNHS. **Method S2**. 16S rRNA gene sequencing bioinformatics in GNHS. **Method. S3** Targeted fecal metabolomics profiling in GNHS. **Method S4**. Fruit/vegetable-microbiota index calculation. **Table S1.** Characteristics of study participants from the GGMP. **Table S2.** The detailed items in the fruit and vegetable categories. **Table S3.** Characteristics of study participants by total fruit and vegetable intake from the GNHS. **Table S4.** Characteristics of study participants by sex from the GNHS. **Table S5.** Gut microbiota biomarkers of fruit intake comparing quartile 4 with quartile 1. **Table S6.** Gut microbial biomarkers of vegetable intake comparing quartile 4 with quartile 1. **Table S7.** Gut microbial biomarkers of total fruit and vegetable intake comparing quartile 4 with quartile 1. **Table S8.** Sensitivity analysis for the relationships among the fruit intake, fruit-microbiota index and type 2 diabetes in the GGMP. **Figure S1.** The prospective associations of vegetable intake and total fruit and vegetable intake with α-diversity. **Figure S2.** The prospective associations of vegetable intake and total fruit and vegetable intake with β-diversity. **Figure S3.** The association of fruit-microbiota index with fruit categories in the Guangzhou Nutrition and Health Study. **Figure S4.** Relationships between the fruit intake-associated gut microbiota alterations and T2D-related traits. **Figure S5.** Associations of the vegetable/total fruit and vegetable intake-associated gut microbiota alterations with corresponding dietary intake and T2D. **Figure S6.** Association of the fruit-associated gut microbiota index and fecal metabolites. **Figure S7.** Fruit intake-related gut microbiota, fecal metabolites and T2D. **Figure S8.** Pathways enrichment analysis of the identified fecal metabolites associated with the fruit-related gut microbiota.

## Data Availability

Data of the present research is available from the corresponding author on reasonable request. For the Guangzhou Nutrition and Health Study (GNHS), the raw data for 16S rRNA gene sequences are available in the CNSA (https://db.cngb.org/cnsa/) of CNGBdb at accession number CNP0000829. For the Guangdong Gut Microbiome Project (GGMP), the raw data for 16S rRNA gene sequences are available from the European Nucleotide Archive (https://www.ebi.ac.uk/ena) at accession number PRJEB18535.
