# Supplementary material for: Dietary fruit and vegetable intake, gut microbiota, and type 2 diabetes: results from two large human cohort studies
Source: BMC Med. 2020 Dec 3;18:371. doi: 10.1186/s12916-020-01842-0 (PMC7712977; doi:10.1186/s12916-020-01842-0)

**Supplementary** **materials**

**“Dietary fruit and vegetable intake, gut microbiota and type 2 diabetes: results from two large human cohort studies”**

**This supplementary file contains the following:**

**Supplementary Methods：**

**Method S1** Fecal microbial DNA extraction and 16S rRNA gene sequencing in GNHS

**Method S2** 16S rRNA gene sequencing bioinformatics in GNHS

**Method S3** Targeted fecal metabolomics profiling in GNHS

**Method S4** Fruit/vegetable-microbiota index calculation

**Supplementary Tables:**

Table S1 Characteristics of study participants from the GGMP

Table S2 The detailed items in the fruit and vegetable categories

Table S3 Characteristics of study participants by total fruit and vegetable intake from the GNHS

Table S4 Characteristics of study participants by sex from the GNHS

Table S5 Gut microbiota biomarkers of fruit intake comparing quartile 4 with quartile 1

Table S6 Gut microbial biomarkers of vegetable intake comparing quartile 4 with quartile 1

Table S7 Gut microbial biomarkers of total fruit and vegetable intake comparing quartile 4 with quartile 1

Table S8 Sensitivity analysis for the relationships among the fruit intake, fruit-microbiota index and type 2 diabetes in the GGMP

**Supplementary Figures:**

**Figure S1 The prospective associations of vegetable intake and total fruit and vegetable intake with α-diversity.** (A) α-diversity for vegetable intake: Observed species, Chao 1’s diversity parameter, Shannon’s diversity parameter, adjusted for Bristol stool score, sequencing run, sequencing depth, age, sex, BMI, smoking status, alcohol status, physical activity, education, income, T2D status, drug use (medications for hypertension, hyperlipidemia and T2D), total energy intake, fruit intake, red and processed meat, fish and dairy products. (B) α-diversity for total fruit and vegetable intake, adjusted for Bristol stool score, sequencing run, sequencing depth, age, sex, BMI, smoking status, alcohol status, physical activity, education, income, T2D status, drug use (medications for hypertension, hyperlipidemia and T2D), total energy intake, red and processed meat, fish and dairy products. Multivariable linear regression was used to estimate the difference in α-diversity comparing extreme quartiles of vegetable intake and total fruit and vegetable intake respectively. Value with “ns” represents no significant difference and asterisk is significantly different (**p* < 0.05, ** *p* < 0.01, *** *p* < 0.001).

**Figure S2 The prospective associations of vegetable intake and total fruit and vegetable intake with β-diversity.** Principal coordinate analysis (PCoA) plot based on Bray-Cutis distance at operational taxonomic unit (OTU) level. Permutational ANOVA (PERMANOVA) (999 permutations) was used to identify the variation of β-diversity in human gut microbiota structure comparing extreme quartiles of (A) vegetable intake, adjusted for Bristol stool score, sequencing run, sequencing depth, age, sex, BMI, smoking status, alcohol status, physical activity, education, income, T2D status, drug use (medications for hypertension, hyperlipidemia and T2D), total energy intake, fruit intake, red and processed meat, fish and dairy products, and (B) total fruit and vegetable intake, adjusted for Bristol stool score, sequencing run, sequencing depth, age, sex, BMI, smoking status, alcohol status, physical activity, education, income, T2D status, drug use (medications for hypertension, hyperlipidemia and T2D), total energy intake, red and processed meat, fish and dairy products, respectively. Value with asterisk is significantly different (**p* < 0.05, ** *p* < 0.01, ****p* < 0.001).

**Figure S3 The association of fruit-microbiota index with fruit categories in the Guangzhou Nutrition and Health Study.** Violin plot showing the association (partial correlation analysis) of the fruit-microbiota index with fruit categories adjusted for age, sex and BMI as confounders. The x axis shows the partial correlation rho values and the y axis indicates the -log (base 10) of the *p* values. Points are coloured based on the significance of the obtained associations (red indicates associations with *p*<0.05). The Benjamini-Hochberg method was used to adjust *p* values for multiple testing.

**Figure S4 Relationships between the fruit intake-associated gut microbiota alterations and T2D-related traits.** Associations of fruit-microbiota index (FMI) (per standardized unit increase) with T2D-related traits (fasting serum insulin, glucose, glycated hemoglobin (HbAlc), homeostasis model assessment of insulin resistance (HOMA-IR) and β-cell function (HOMA-β) (per standardized unit)) in the Guangzhou Nutrition and Health Study (GNHS), respectively, adjusted for Bristol stool score, sequencing run, sequencing depth, age, sex, BMI, smoking status, alcohol status, physical activity, education, income, T2D status, drug use (medications for hypertension, hyperlipidemia and T2D), total energy intake, vegetable intake, red and processed meat, fish and dairy products. *p* value < 0.05 is significantly different.

**Figure S5 Associations of the vegetable/total fruit and vegetable intake-associated gut microbiota alterations with corresponding dietary intake and T2D.** Associations of vegetable-microbiota index (FMI) and total fruit and vegetable-microbiota index (TFAMI) with corresponding dietary intake (A) and T2D (B) in the Guangzhou Nutrition and Health Study (GNHS), respectively. *p* value < 0.05 is significantly different.

**Figure S6 Association of the fruit-associated gut microbiota index and fecal metabolites.** Violin plot showing the association (partial correlation analysis) of the fruit-microbiota index (FMI) with fecal metabolic profiles, adjusted for age, sex and BMI. The x axis shows the partial correlation rho values and the y axis indicates the -log (base 10) of the *p* values. Points are coloured based on the significance of the obtained associations (red indicates positive associations with *p*<0.05, green indicates negative associations with *p*<0.05). The Benjamini-Hochberg method was used to adjust *p* values for multiple testing.

**Figure S7 Fruit intake-related gut microbiota, fecal metabolites and T2D. (A)** Heatmap showing the variation of the association patterns of the fruit-associated gut microbiome alterations and specific fecal metabolites based on partial correlation analysis adjusted for age, sex and BMI. For each cell, colours indicate the partial correlation rho values (red indicates positive associations with *p*<0.05, blue indicates negative associations with *p*<0.05). The identified 76 fecal metabolites were clustered into three modules. The module 1 represent the metabolites showing significantly positive association with the fruit-associated gut microbiota. The module 2 and 3 showing the metabolites significantly negative association with the fruit-associated gut microbiota. **(B)** Co-occurrence network between the fruit-associated gut microbiota and specific fecal metabolites. The network shows two distinct characteristics of the fruit-positive and fruit-negative gut microbiota on modulating the fecal metabolites (red circles indicate fruit-positive OTUs, green circles indicate fruit-negative OTUs, red lines indicate positive association, and green lines indicate negative association). **(C)** Inter-relationships among the fruit-related gut microbiota, fecal metabolites and T2D (red metabolite circles indicate T2D-positive metabolites, green metabolite circles indicate T2D-negative metabolites, green OTU circles indicate fruit-positive OTUs, red OTU circles indicate fruit-negative OTUs, red lines indicate positive association, and green lines indicate negative association).

**Figure S8 Pathways enrichment analysis of the identified fecal metabolites associated with the fruit-related gut microbiota.** Pathways enrichment analyses of the identified fecal metabolites were performed by MetaboAnalyst 4.0. Points are coloured based on the significance of the obtained associations (red indicates FMI-positive association with *p*<0.05, green indicates FMI-negative association with *p*<0.05). Partial correlation analysis was used to calculate the association of the fruit-microbiota index with fecal metabolites, adjusted for age, sex and BMI. *p* value < 0.05 is significantly different. The Benjamini-Hochberg method was used to adjust *p* values for multiple testing.**Supplemental Methods**

**Method S1 Fecal microbial DNA extraction and 16S rRNA gene sequencing in GNHS**

Fecal microbial DNA was extracted from each sample using the QIAamp® DNA Stool Mini Kit (Qiagen, Hilden, Germany) following the manufacturer’s instruction. DNA concentration and purity were monitored on 1% agarose gels. According to the concentration, DNA was diluted to 1 ng/μL using sterile water. The 16S rRNA gene amplification procedure was divided into two PCR steps, in the first PCR reaction, the V3-V4 hypervariable region of the 16S rRNA gene was amplified from genomic DNA using primers 341F(CCTACGGGNGGCWGCAG) and 805R(GACTACHVGGGTATCTAATCC). Amplification was performed in 96-well microtiter plates with a reaction mixture consisting of 1X KAPA HiFi Hot start Ready Mix, 0.1µM primer 341 F, 0.1 µM primer 805 R, and 12.5 ng template DNA, giving a total volume of 50 µL per sample. Reactions were run in a T100 PCR thermocycle (BIO-RAD) according to the following cycling program: 3 min of denaturation at 94 °C, followed by 18 cycles of 30 s at 94 °C (denaturing), 30 s at 55 °C (annealing), and 30 s at 72 °C (elongation), with a final extension at 72 °C for 5 min. Subsequently, the amplified products were checked by 2% agarose gel electrophoresis and ethidium bromide staining. Amplicons were quantified using the Qubit quantification system (Thermo Scientific, Wilmington, DE, US) following the manufacturers’ instructions. Sequencing primers and adaptors were added to the amplicon products in the second PCR step as follows 2 µL of the diluted amplicons were mixed with a reaction solution consisting of 1×KAPA HiFi Hotstart ReadyMix, 0.5µM fusion forward and 0.5µM fusion reverse primer, 30 ng Meta-gDNA(total volume 50 µL). The PCR was run according to the cycling program above except with cycling number of 12. The amplification products were purified with Agencourt AMPure XP Beads (Beckman Coulter Genomics, MA, USA) according to the manufacturer’s instructions and quantified as described above. Equimolar amounts of the amplification products were pooled together in a single tube. The concentration of the pooled libraries was determined by the Qubit quantification system. Amplicon sequencing was performed on the Illuimina MiSeq System (Illumina Inc., CA, USA). The MiSeq Reagent Kits v2 (Illumina Inc.) was used. Automated cluster generation and 2 × 250 bp paired-end sequencing with dual-index reads were performed.

**Method S2 16S rRNA gene sequencing bioinformatics in GNHS**

Fastq-files were demultiplexed by the MiSeq Controller Software (Illumina Inc.). The sequences were trimmed for amplification primers, diversity spacers, and sequencing adapters, merge-paired and quality filtered by USEARCH. UPARSE was used for operational taxonomic unit (OTU) clustering equaling or above 97%. Taxonomy of the OTUs was assigned and sequences were aligned with RDP classifier. A representative sequence was picked for each OTU and the Greengenes reference database was used to annotate taxonomic information for each representative sequence. The OTUs were analyzed by phylogenetic and operational taxonomic unit methods in the Quantitative Insights into Microbial Ecology (QIIME) software version 1.9.0. α-diversity (Observed species, Shannon index, Chao1 index) and β-diversity (Bray-Curtis distance) measures were calculated based on the rarefied OTU counts. The OTU absolute abundance table was extracted from the pipeline and converted to relative abundances by normalizing to total OTU clustering for analyzing the composition of gut microbiota.

Principal coordinate analysis (PCoA) based on Bray-Curtis distance and permutational multivariate analysis of variance (PERMANOVA) were performed to compare the dissimilarity of global microbiota composition. MaAsLin is a multivariate statistical framework that identifies associations between continuous and discrete clinical metadata and microbial community abundances. Microbiome-based biomarker discovery was performed with MaAsLin using the online galaxy server (<https://huttenhower.sph.harvard.edu/galaxy/>). Briefly, the percentage of each OTU was arcsin-square-root-transformed. For each correlation between metadata (as predictors) and transformed OTU abundances (as response variables), we adjusted potential confounders including Bristol stool score, sequencing run, sequencing depth, age, sex, BMI, smoking status, alcohol status, physical activity, education, income, T2D status, drug use (medications for hypertension, hyperlipidemia and T2D), total energy intake, dietary intake of vegetable /fruit (mutual adjustment for each other), red and processed meat, fish and dairy products. To further reduce the computational load, OTUs that were present in < 10% of the population were excluded. The Benjamini-Hochberg method was used to adjust *p* values for multiple hypotheses.

**Method S3 Targeted fecal metabolomics profiling in GNHS**

The targeted metabolomics profiling of fecal samples (n=1017) was performed by Metabo-Profile (Shanghai, China). The order of all test samples is randomly selected before the preparation. 10 mg lyophilized feces were homogenized with 25μL water and extracted with 185μL cold ACN-Methanol (8/2, v/v). After centrifugation, 30 μL supernatant was used to derivatization with 20 μL freshly prepared derivative reagents on a Biomek 4000 workstation (Biomek 4000, Beckman Coulter, Inc., Brea, California, USA), followed by mixing with internal standards. Subsequently, the derivatized samples and serial dilutions of derivatized stock standards were randomly analyzed and quantitated by an ultra-performance liquid chromatography coupled to tandem mass spectrometry (UPLC-MS/MS) system (ACQUITY UPLC-Xevo TQ-S, Waters Corp., Milford, MA, USA). 310 of standard substances including 12 subclasses were obtained from Sigma-Aldrich (St. Louis, MO, USA), Steraloids Inc. (Newport, RI, USA) and TRC Chemicals (Toronto, ON, Canada). Three types of quality control samples i.e., test mixtures, internal standards, and pooled biological samples are routinely used in metabolomics platform. The derivatized pooled quality control samples were injected every 14 test samples. Raw data generated by UPLC-MS/MS were processed using the QuanMET software (v2.0, Metabo-Profile, Shanghai, China) to perform peak integration, calibration, and quantification for each metabolite.

**Method S4 Fruit/vegetable-microbiota index calculation**

To summarize the association of fruit and vegetable on the gut microbes, we calculated fruit-microbiota index (FMI), vegetable-microbiota index (VMI) and total fruit and vegetable-microbiota index (TFVMI) based on the identified OTUs for each of the three dietary variables, detailed in Supplemental methods.

The indices were calculated as the formula below:

$I_{i}^{P}=\sum_{j=1}^{n} A_{ij}$ $I_{i}^{N}=\sum_{j=1}^{m} A_{ij}$ $X_{i}=\left( \frac{I_{i}^{P}}{n}-\frac{I_{i}^{N}}{m} \right)\times10000$

$$FMI/VMI/TFVMI=\frac{X_{i}-\bar{X}}{\mathrm{SD}}$$

where $A_{ij}$is the relative abundance of an OUT $j$ (identified from MaAsLin for the individual $i$, Supplementary Table 3, 4 and 5). *P* is a subset of all positively correlated OUT biomarkers in these gut microbiota. *N* is a subset of all negatively correlated OUT biomarkers in these gut microbiota. $\bar{X}$ is the mean value of $X_{i}$. SD is the is the standard deviation of $X_{i}.$Only those OTUs that were significantly correlated with fruit intake, vegetable intake or total fruit and vegetable intake were used in the calculation.

**Supplementary Tables**

**Table S1 Characteristics of study participants from the GGMP***

| Characteristics | Total population |
| --- | --- |
| n | 6626 |
| Age, y | 52.8 (14.7) |
| Sex, n (% of women) | 3657 (55.2) |
| BMI, kg/m^2^ | 23.4 (3.5) |
| Vegetable intake, g/d | 337 (230) |
| Fruit intake, g/d | 79 (117) |
| Total fruit and vegetable intake, g/d | 416 (272) |
| Red and processed meat intake, g/d | 131 (134) |
| Current alcohol drinker, n (%) | 2606 (39.3) |
| Current smoker, n (%) | 1715 (25.9) |
| Education, n (%) |  |
| Middle school or lower | 5036 (76.0) |
| High school or professional college | 1316 (19.9) |
| University | 274 (4.1) |

*****Data are expressed as mean (SD) for continuous variables and n (%) for categorical variables.

**Table S2 The detailed items in the fruit and vegetable categories**

|  | Fruit items |  | Vegetable items |
| --- | --- | --- | --- |
| 1 | Oranges, tangerines, pomelos, oranges, etc | 1 | Flowering cabbage, mustard and broccoli |
| 2 | Apple, pear, peach, plum, pineapple, etc | 2 | Pakchoi cabbage, lettuces and lettuce |
| 3 | Bananas | 3 | Spinach, amaranth and water spinach |
| 4 | Grapes and raisins | 4 | Other dark green vegetables: leeks, garlic leaves, asparagus, etc |
| 5 | Lychee and longan | 5 | Chinese cabbage, white Cabbage, cauliflower and celery |
| 6 | Mango and persimmon | 6 | Onions, scallions and garlic |
| 7 | pawpaw | 7 | White gourd, cucumber, towel gourd, eggplant, pumpkin, balsam pear, white radish and green radish |
| 8 | Cantaloupe, watermelon and melon | 8 | Tomato |
| 9 | Durian | 9 | Green, red, and bell peppers |
| 10 | The other fruits | 10 | Carrots |
|  |  | 11 | Starchy vegetables: potato, sweet potato, taro, lotus root, water chestnut, kudzu, etc |
|  |  | 12 | Millet and corn |
|  |  | 13 | Sauerkraut, pickles, pickled mustard, preserved vegetable, etc |

**Table S3 Characteristics of study participants by total fruit and vegetable intake from the GNHS***

| Characteristics | Total fruit and vegetable intake | | | | | |
| --- | --- | --- | --- | --- | --- | --- |
|  | Q1 | Q2 | | Q3 | Q4 | *p*-trend |
| n | 470 | 470 | | 470 | 469 |  |
| Age, y | 59.0 (7.1) | 58.9 (5.9) | 58.4 (5.7) | | 58.1 (5.5) | 0.009 |
| Sex, n (% of women) | 276 (58.7) | 303 (64.5) | | 338 (71.9) | 347 (74.0) | <0.001 |
| BMI, kg/m^2^ | 23.3 (3.2) | 23.2 (2.9) | | 23.1 (2.8) | 23.3 (3.1) | 0.557 |
| Total energy intake, kcal/d | 1494 (425) | 1654 (431) | | 1830 (451) | 1992 (494) | <0.001 |
| Physical activity, MET hours/d | 38.8 (12.9) | 39.2 (13.8) | | 41.1 (14.0) | 43.3 (15.1) | <0.001 |
| Vegetable intake, g/d | 205 (56) | 314 (62) | | 416 (79) | 600 (186) | <0.001 |
| Fruit intake, g/d | 66 (41) | 112 (57) | | 157 (76) | 248 (136) | <0.001 |
| Total fruit and vegetable intake, g/d | 271 (59) | 426 (42) | | 573 (47) | 848 (202) | <0.001 |
| Red and processed meat intake, g/d | 90 (55) | 100 (57) | | 109 (60) | 117 (69) | <0.001 |
| Fish intake, g/d | 37 (55) | 45 (43) | | 55 (59) | 64 (43) | <0.001 |
| Dairy products intake, g/d | 90 (102) | 105 (101) | | 128 (117) | 138 (129) | <0.001 |
| Current alcohol drinker, n (%) | 45 (9.6) | 37 (7.9) | | 26 (5.5) | 29 (6.2) | 0.020 |
| Current smoker, n (%) | 107 (22.8) | 72 (15.3) | | 58 (12.3) | 55 (11.7) | <0.001 |
| Income level, n (%) | | | | | | 0.109 |
| ≤ 500 ¥/mo | 6 (1.3) | 6 (1.3) | | 10 (2.1) | 6 (1.3) |  |
| 501-1500 ¥/mo | 91 (19.4) | 101 (21.5) | | 112 (23.8) | 99 (21.1) |  |
| 1501-3000 ¥/mo | 336 (71.5) | 302 (64.3) | | 275 (58.5) | 284 (60.6) |  |
| > 3000 ¥/mo | 37 (7.9) | 61 (13.0) | | 73 (15.5) | 80 (17.1) |  |
| Education, n (%) | | | | | | 0.790 |
| Middle school or lower | 133 (28.3) | 125 (26.6) | | 134 (28.5) | 118 (25.2) |  |
| High school or professional college | 206 (43.8) | 208 (44.3) | | 218 (46.4) | 232 (49.4) |  |
| University | 131 (27.9) | 137 (29.1) | | 118 (25.1) | 119 (25.4) |  |
| Glucose, mmol/L | 5.49 (1.27) | 5.47 (1.37) | | 5.50 (1.42) | 5.46 (1.20) | 0.796 |
| Insulin, μU/mL | 7.58 (4.42) | 7.37 (4.13) | | 7.19 (3.84) | 7.03 (3.49) | 0.045 |
| HbAlc, % | 7.12 (4.00) | 7.15 (4.50) | | 7.41 (4.21) | 7.28 (4.16) | 0.407 |
| HOMA-IR | 1.90 (1.34) | 1.83 (1.25) | | 1.81 (1.19) | 1.77 (1.10) | 0.136 |
| HOMA-β, % | 89.2 (60.7) | 88.1 (59.5) | | 81.4 (47.8) | 81.5 (45.8) | 0.017 |
| Medication use, n (%) | | | | | | 0.262 |
| Hypertension | 19 (4.0) | 27 (5.7) | | 19 (4.0) | 35 (7.5) |  |
| Hyperlipidaemia | 39 (8.3) | 23 (4.9) | | 27 (5.7) | 22 (4.7) |  |
| T2D | 21 (4.5) | 15 (3.2) | | 11 (2.3) | 11 (2.3) |  |

*****Data are expressed as mean (SD) for continuous variables and n (%) for categorical variables; Q1: quartile 1, Q2: quartile 2, Q3: quartile 3 and Q4: quartile 4; Q1 indicates the quartile with the lowest intake; *p*-trend represents the comparison among quartiles using linear regression; HbAlc: glycated hemoglobin, HOMA-IR: homeostasis model assessment of insulin resistance, HOMA-β: homeostasis model assessment of β-cell function, T2D: type 2 diabetes.

**Table S4 Characteristics of study participants by sex from the GNHS***

| Characteristics | Men | Women | *p*-value |
| --- | --- | --- | --- |
| n | 615 | 1264 |  |
| Age, y | 60.4 (6.6) | 57.7 (5.6) | <0.001 |
| BMI, kg/m^2^ | 23.7 (2.7) | 23.0 (3.1) | <0.001 |
| Total energy intake, kcal/d | 1898 (530) | 1667 (447) | <0.001 |
| Physical activity, MET hours/d | 39.6 (13.8) | 41.1 (14.2) | 0.033 |
| Vegetable intake, g/d | 370 (192) | 390 (177) | 0.025 |
| Fruit intake, g/d | 123 (92) | 157 (115) | <0.001 |
| Total fruit and vegetable intake, g/d | 493 (238) | 547 (248) | <0.001 |
| Red and processed meat intake, g/d | 112 (63) | 101 (60) | <0.001 |
| Fish intake, g/d | 50 (37) | 50 (57) | 0.980 |
| Dairy products intake, g/d | 90 (102) | 128 (117) | <0.001 |
| Current alcohol drinker, n (%) | 103 (16.7) | 34 (2.7) | <0.001 |
| Current smoker, n (%) | 287 (46.7) | 5 (0.4) | <0.001 |
| Income level, n (%) | | | 0.026 |
| ≤ 500 ¥/mo | 11 (1.8) | 17 (1.3) |  |
| 501-1500 ¥/mo | 110 (17.9) | 293 (23.2) |  |
| 1501-3000 ¥/mo | 418 (68.0) | 779 (61.6) |  |
| > 3000 ¥/mo | 76 (12.4) | 175 (13.8) |  |
| Education, n (%) | | | <0.001 |
| Middle school or lower | 151 (24.6) | 359 (28.4) |  |
| High school or professional college | 237 (38.5) | 627 (49.6) |  |
| University | 227 (36.9) | 278 (22.0) |  |
| Glucose, mmol/L | 5.56 (1.38) | 5.44 (1.28) | 0.054 |
| Insulin, μU/mL | 6.89 (3.47) | 7.51 (4.24) | 0.003 |
| HbAlc, % | 7.00 (3.30) | 7.35 (4.60) | 0.105 |
| HOMA-IR | 1.74 (1.06) | 1.88 (1.31) | 0.031 |
| HOMA-β, % | 81.1 (54.7) | 87.4 (54.0) | 0.032 |
| Medication use, n (%) | | | 0.152 |
| Hypertension | 35 (5.7) | 65 (5.1) |  |
| Hyperlipidaemia | 38 (6.2) | 73 (5.8) |  |
| T2D | 24 (3.9) | 34 (2.7) |  |

*****Data are expressed as mean (SD) for continuous variables and n (%) for categorical variables; *p-*value represents the comparison between sex using independent t-test or chi square; HbAlc: glycated hemoglobin, HOMA-IR: homeostasis model assessment of insulin resistance, HOMA-β: homeostasis model assessment of β-cell function, T2D: type 2 diabetes.

**Table S5 Gut microbiota biomarkers of fruit intake comparing quartile 4 with quartile 1***

| OTUs | Coefficient | Correlation direction | Q.value | Phylum | Family | Genus | Species |
| --- | --- | --- | --- | --- | --- | --- | --- |
| OTU33187 | 0.041 | Positive | 0.026 | Firmicutes | Ruminococcaceae | Faecalibacterium | prausnitzii |
| OTU12028 | 0.022 | Positive | 0.037 | Firmicutes | Ruminococcaceae | Ruminococcaceae spp1 |  |
| OTU17395 | 0.093 | Positive | 0.047 | Firmicutes | Ruminococcaceae | Ruminococcaceae spp2 |  |
| OTU175436 | 0.048 | Positive | 0.003 | Firmicutes | Ruminococcaceae | Ruminococcaceae spp3 |  |
| OTU176290 | 0.025 | Positive | 0.025 | Firmicutes | Ruminococcaceae | Ruminococcaceae spp4 |  |
| OTU17847 | 0.109 | Positive | 0.011 | Firmicutes | Ruminococcaceae | Ruminococcaceae spp5 |  |
| OTU18 | 0.094 | Positive | 0.041 | Firmicutes | Ruminococcaceae | Ruminococcaceae spp6 |  |
| OTU180674 | 0.015 | Positive | 0.028 | Firmicutes | Ruminococcaceae | Ruminococcaceae spp7 |  |
| OTU190159 | 0.032 | Positive | 0.007 | Firmicutes | Ruminococcaceae | Ruminococcaceae spp8 |  |
| OTU192248 | 0.020 | Positive | .0044 | Firmicutes | Ruminococcaceae | Ruminococcaceae spp9 |  |
| OTU192835 | 0.024 | Positive | 0.026 | Firmicutes | Ruminococcaceae | Ruminococcaceae spp10 |  |
| OTU194679 | 0.034 | Positive | 0.009 | Firmicutes | Ruminococcaceae | Ruminococcaceae spp11 |  |
| OTU214075 | 0.016 | Positive | 0.014 | Firmicutes | Ruminococcaceae | Ruminococcaceae spp12 |  |
| OTU219220 | 0.040 | Positive | 0.004 | Firmicutes | Ruminococcaceae | Ruminococcaceae spp13 |  |
| OTU226324 | 0.023 | Positive | 0.044 | Firmicutes | Ruminococcaceae | Ruminococcaceae spp14 |  |
| OTU23457 | 0.089 | Positive | 0.020 | Firmicutes | Ruminococcaceae | Ruminococcaceae spp15 |  |
| OTU245548 | 0.045 | Positive | 0.010 | Firmicutes | Ruminococcaceae | Ruminococcaceae spp16 |  |
| OTU247346 | 0.024 | Positive | 0.004 | Firmicutes | Ruminococcaceae | Ruminococcaceae spp17 |  |
| OTU255514 | 0.016 | Positive | 0.025 | Firmicutes | Ruminococcaceae | Ruminococcaceae spp18 |  |
| OTU3483 | 0.025 | Positive | 0.039 | Firmicutes | Ruminococcaceae | Ruminococcaceae spp19 |  |
| OTU52 | 0.020 | Positive | 0.025 | Firmicutes | Ruminococcaceae | Ruminococcaceae spp20 |  |
| OTU90807 | 0.051 | Positive | 0.005 | Firmicutes | Ruminococcaceae | Ruminococcaceae spp21 |  |
| OTU1934 | 0.013 | Positive | 0.028 | Firmicutes | Clostridiales | Clostridiales spp1 |  |
| OTU19446 | 0.094 | Positive | 0.031 | Firmicutes | Clostridiales | Clostridiales spp2 |  |
| OTU105227 | 0.011 | Positive | 0.046 | Firmicutes | Clostridiales | Clostridiales spp3 |  |
| OTU30107 | 0.016 | Positive | 0.044 | Firmicutes | Veillonellaceae | Acidaminococcus |  |
| OTU77005 | 0.019 | Positive | 0.012 | Proteobacteria | Enterobacteriaceae | Enterobacteriaceae spp |  |
| OTU129135 | 0.018 | Positive | 0.037 | Bacteroidetes | Prevotellaceae | Prevotella | copri |
| OTU14373 | 0.009 | Positive | 0.044 | Bacteroidetes | Prevotellaceae | Prevotella | stercorea |
| OTU182442 | 0.011 | Positive | 0.005 | Verrucomicrobia | Verrucomicrobiaceae | Akkermansia | muciniphila |
| OTU2945 | -0.064 | Negative | 0.045 | Fusobacteria | Fusobacteriaceae | Fusobacterium |  |

*The gut microbiota biomarkers were determined by MaAsLin adjusted by Bristol stool score, sequencing run, sequencing depth, age, sex, BMI, smoking status, alcohol status, physical activity, education, income, T2D status, drug use (medications for hypertension, hyperlipidemia and T2D), total energy intake, vegetable intake, red and processed meat, fish and dairy products. **Table S6 Gut microbial biomarkers of vegetable intake comparing quartile 4 with quartile 1***

| OTU | Coefficient | Correlation direction | Q.value | | Phylum | Family | Genus | Species |
| --- | --- | --- | --- | --- | --- | --- | --- | --- |
| OTU236545 | 0.023 | Positive | 0.034 | Firmicutes | | Lachnospiraceae | Lachnospira |  |

*The gut microbiota biomarkers were determined by MaAsLin (multivariate association analysis) adjusted by Bristol stool score, sequencing run, sequencing depth, age, sex, BMI, smoking status, alcohol status, physical activity, education, income, T2D status, drug use (medications for hypertension, hyperlipidemia and T2D), total energy intake, fruit intake, red and processed meat, fish and dairy products.

**Table S7 Gut microbial biomarkers of total fruit and vegetable intake** **comparing quartile 4 with quartile 1***

| OTUs | Coefficient | | Correlation direction | Q.value | Phylum | Family | Genus | Species |
| --- | --- | --- | --- | --- | --- | --- | --- | --- |
| OTU236545 | | 0.024 | Positive | 0.036 | Firmicutes | Lachnospiraceae | Lachnospira |  |
| OTU234983 | | 0.001 | Positive | 0.043 | Firmicutes | Lachnospiraceae | Lachnospiraceae spp |  |

*The gut microbiota biomarkers were determined by MaAsLin (multivariate association analysis) adjusted by Bristol stool score, sequencing run, sequencing depth, age, sex, BMI, smoking status, alcohol status, physical activity, education, income, T2D status, drug use (medications for hypertension, hyperlipidemia and T2D), total energy intake, red and processed meat, fish and dairy products.**Table S8 Sensitivity analysis for the relationships among the fruit intake, fruit-microbiota index and type 2 diabetes in the GGMP***

| Fruit-microbiota index | Relationships | | | |
| --- | --- | --- | --- | --- |
|  | n | *β* coefficient or Odds ratio | 95%CI | *p* value |
| Fruit intake | 4109 | 3.38 | [0.11, 6.64] | 0.042 |
| T2D risk | 4109 | 0.90 | [0.83, 0.99] | 0.020 |

* Income data is available for 4109 participants; Multivariable linear regression (*β* coefficient) was used to estimate the associations of fruit intake with fruit-microbiota index (FMI); Multivariable logistic regression (Odds ratio) was used to estimate the association of FMI (per standardized unit increase) with type 2 diabetes (T2D) risk (344 cases/4109).

**Supplementary Figures**

**Figure S1 The prospective associations of vegetable intake and total fruit and vegetable intake with α-diversity.**


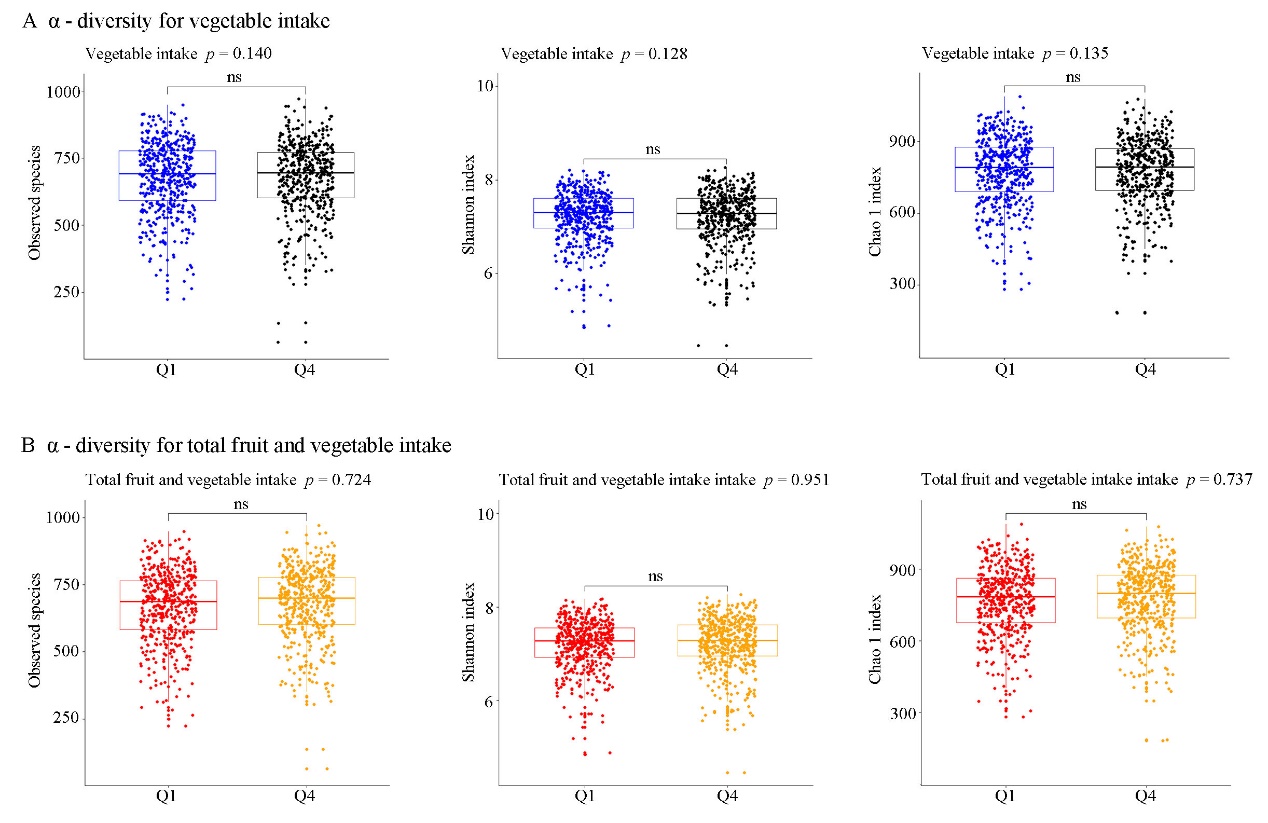


**Figure S2 The prospective associations of vegetable intake and total fruit and vegetable intake with β-diversity.**


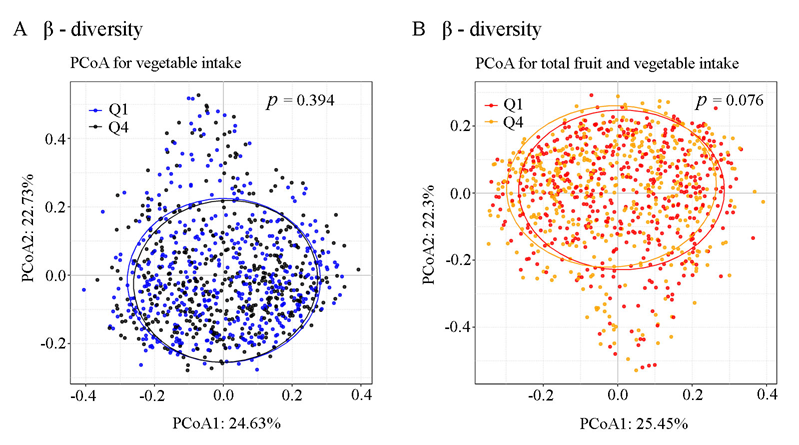


**Figure S3 The association of fruit-microbiota index with fruit categories in the Guangzhou Nutrition and Health Study.**


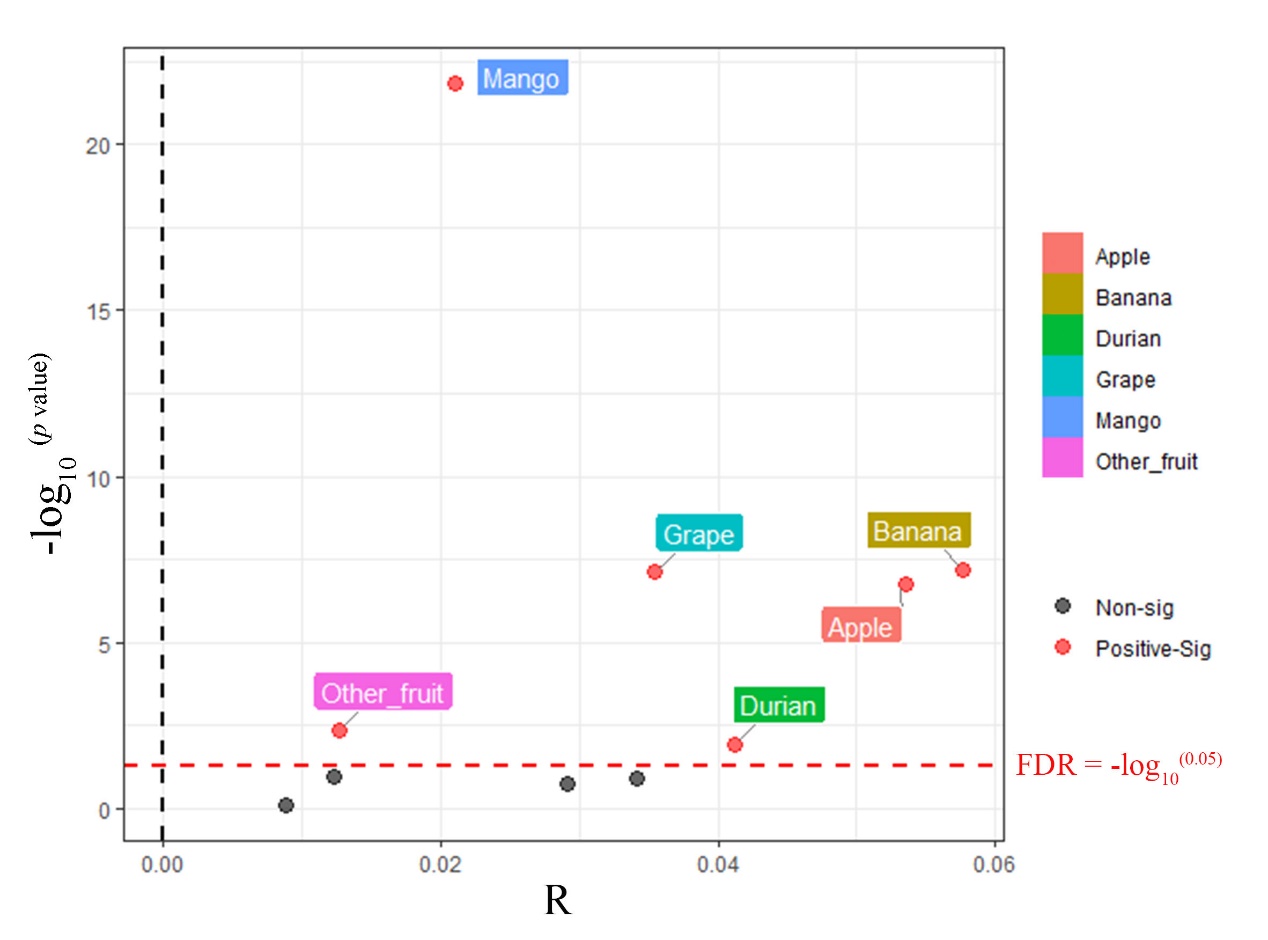
**Figure S4 Relationships between the fruit intake-associated gut microbiota alterations and T2D-related traits.**

**
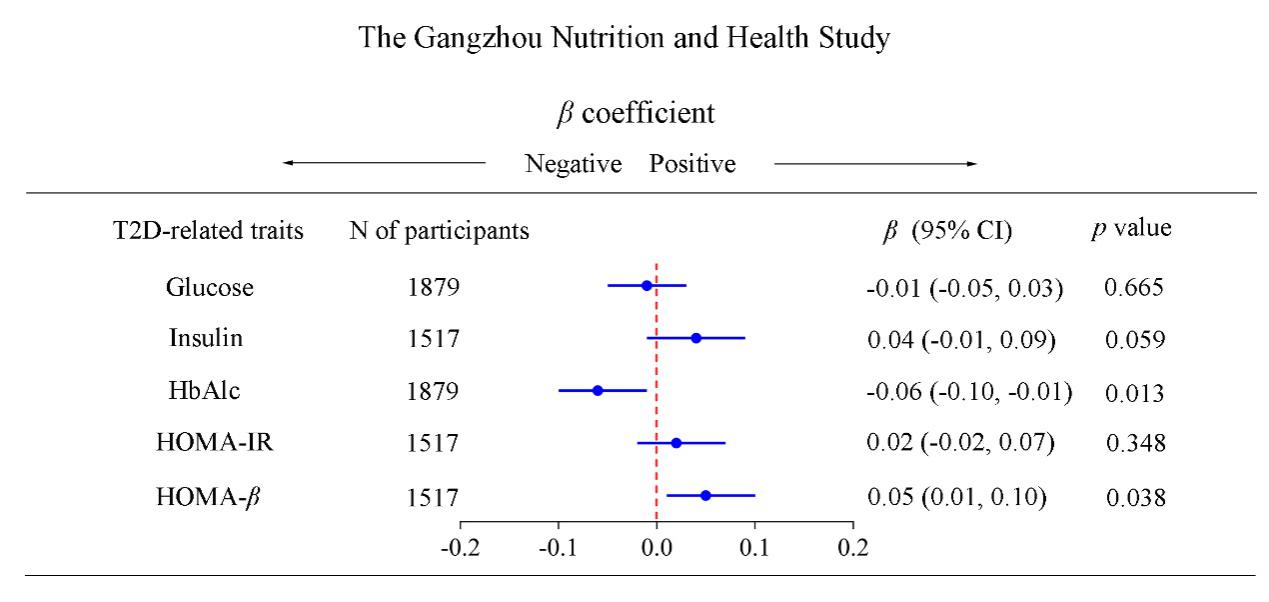
**

**Figure S5 Associations of the vegetable/total fruit and vegetable intake-associated gut microbiota alterations with corresponding dietary intake and T2D.**
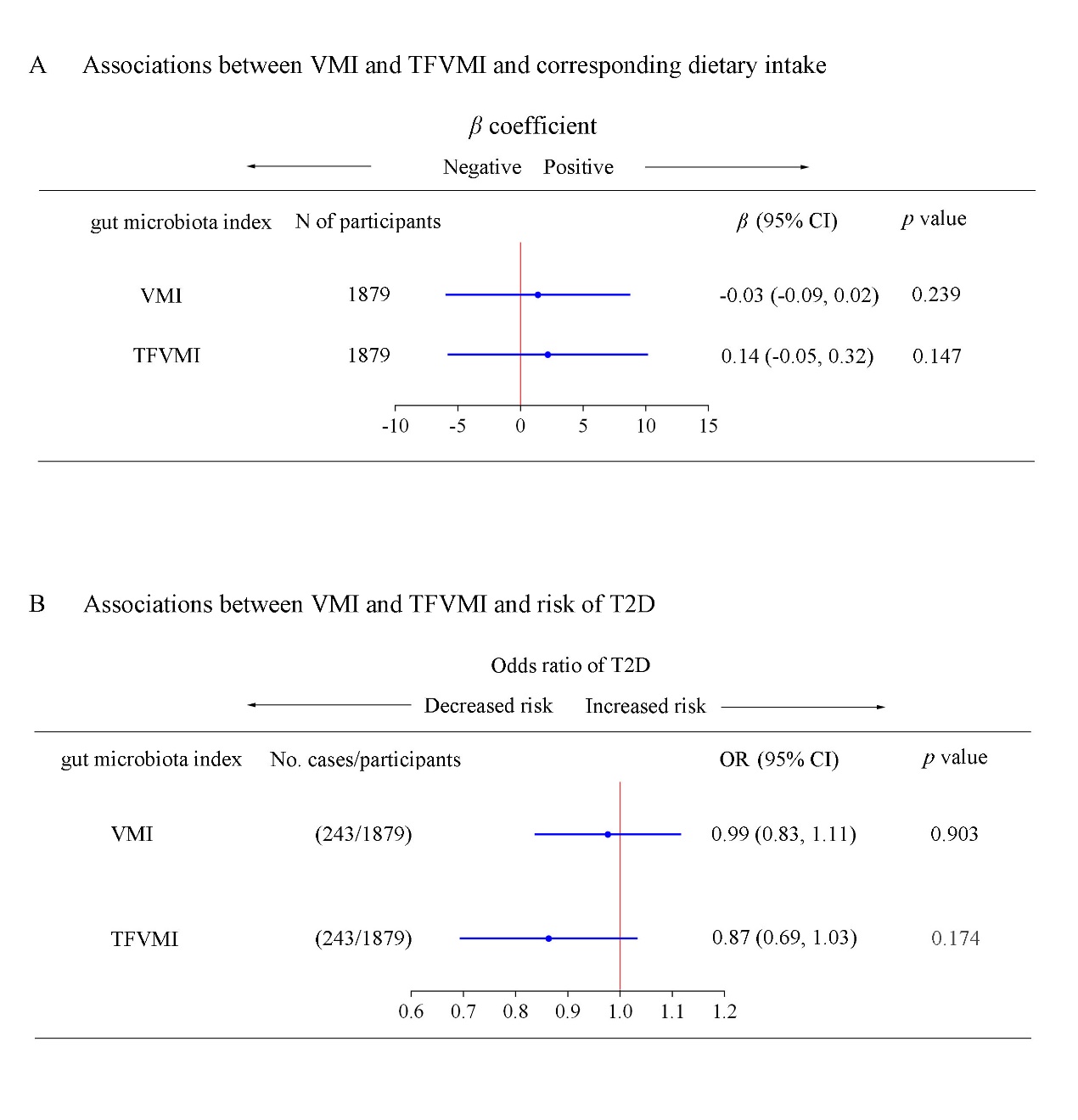


**Figure S6 Association of the fruit-associated gut microbiota index and fecal metabolites.**


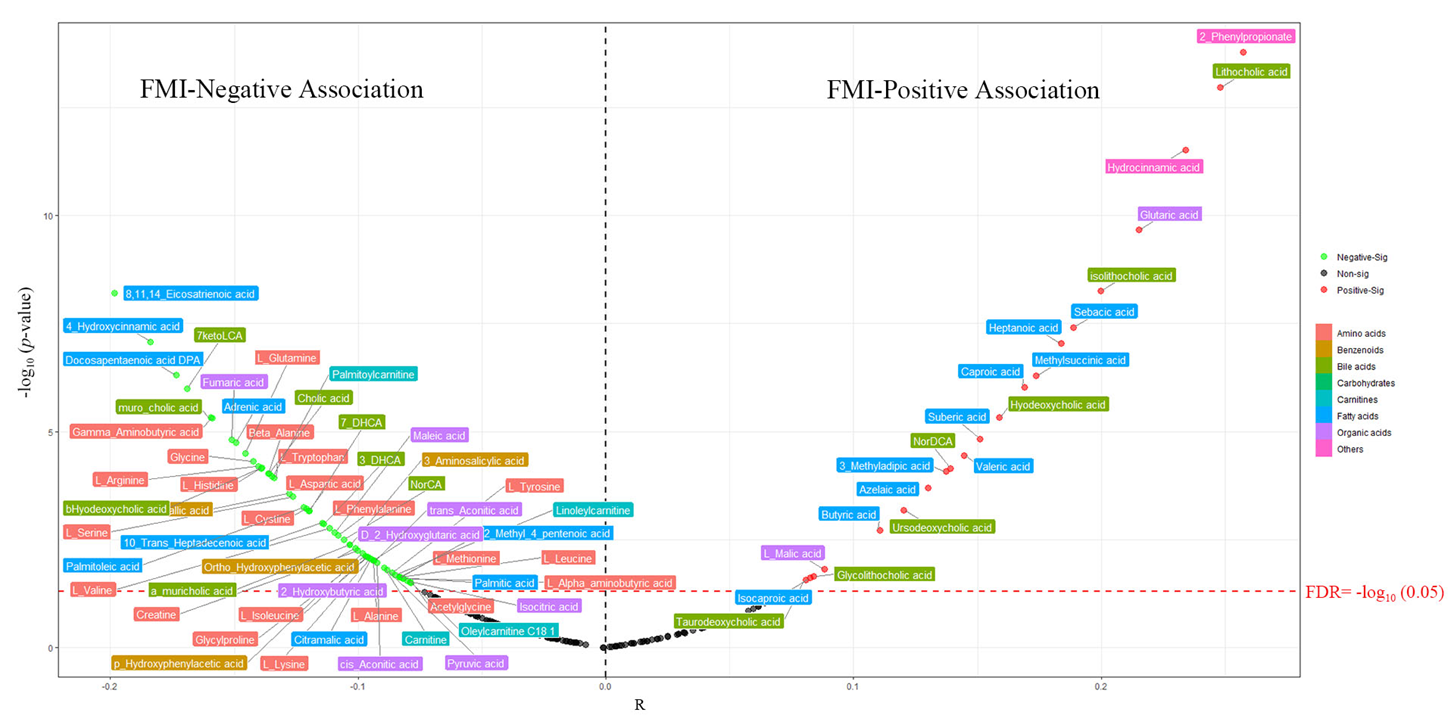


**Figure S7 Fruit intake-related gut microbiota, fecal metabolites and T2D.**


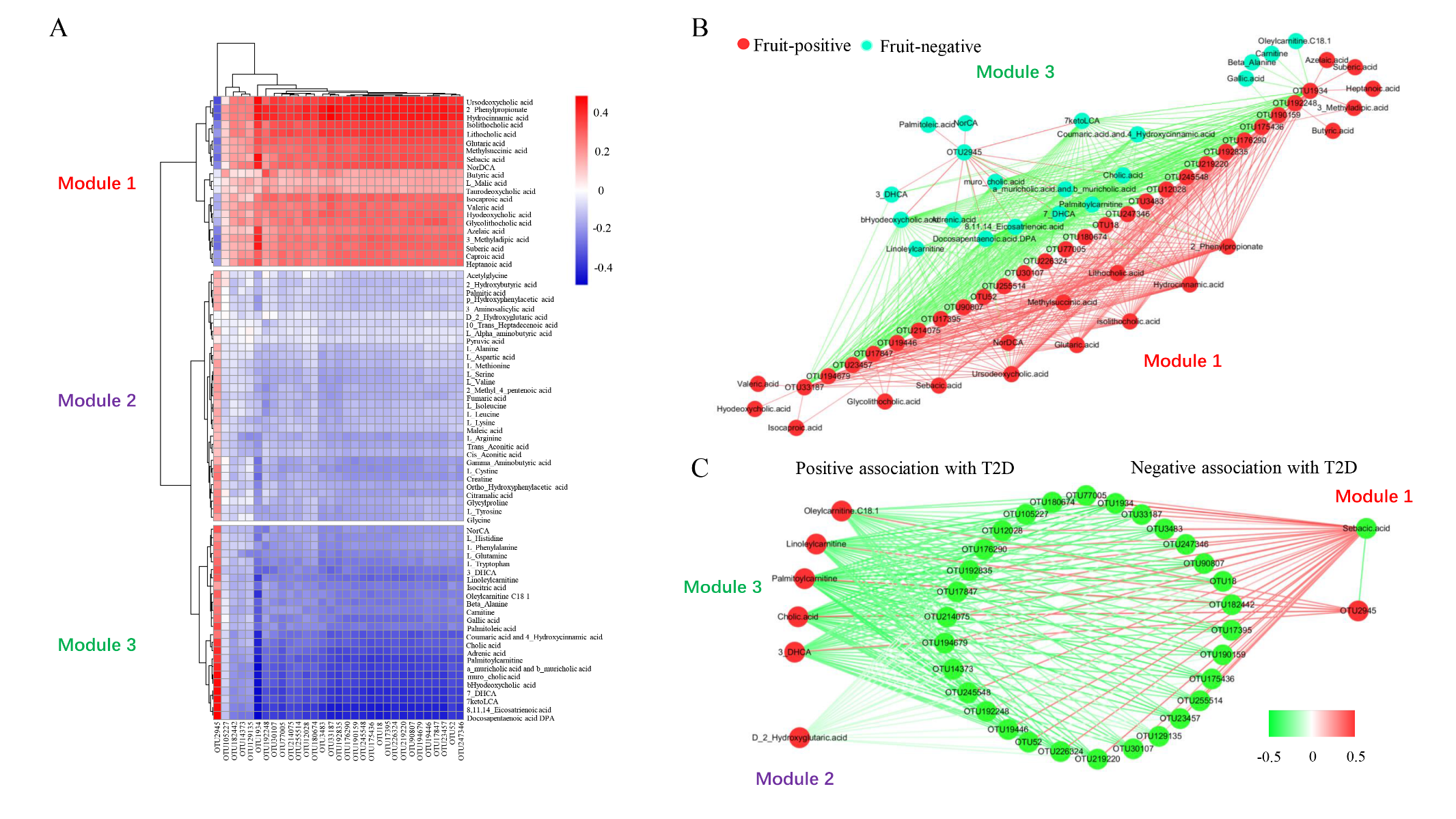


**Figure S8 Pathways enrichment analysis of the identified fecal metabolites associated with the fruit-related gut microbiota.**


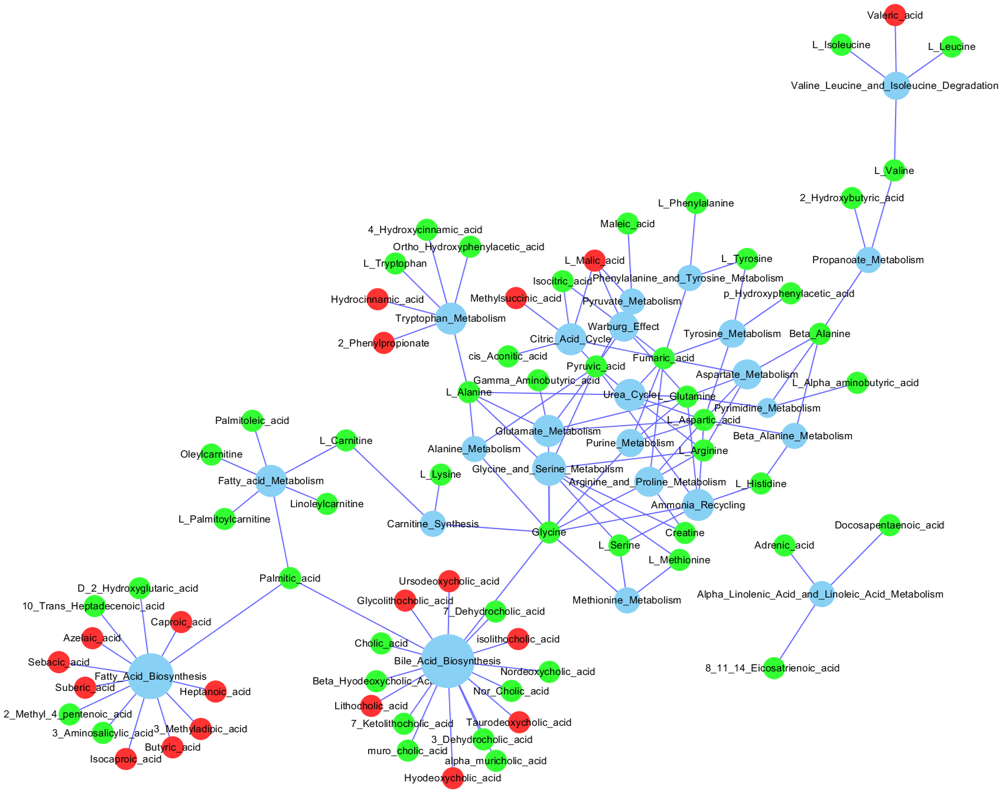

Supplement: Supplementary file 1 — Additional file 1 : Method S1. Fecal microbial DNA extraction and 16S rRNA gene sequencing in GNHS. Method S2. 16S rRNA gene sequencing bioinformatics in GNHS. Method. S3 Targeted fecal metabolomics profiling in GNHS. Method S4. Fruit/vegetable-microbiota index calculation. Table S1. Characteristics of study participants from the GGMP. Table S2. The detailed items in the fruit and vegetable categories. Table S3. Characteristics of study participants by total fruit and vegetable intake from the GNHS. Table S4. Characteristics of study participants by sex from the GNHS. Table S5. Gut microbiota biomarkers of fruit intake comparing quartile 4 with quartile 1. Table S6. Gut microbial biomarkers of vegetable intake comparing quartile 4 with quartile 1. Table S7. Gut microbial biomarkers of total fruit and vegetable intake comparing quartile 4 with quartile 1. Table S8. Sensitivity analysis for the relationships among the fruit intake, fruit-microbiota index and type 2 diabetes in the GGMP. Figure S1. The prospective associations of vegetable intake and total fruit and vegetable intake with α-diversity. Figure S2. The prospective associations of vegetable intake and total fruit and vegetable intake with β-diversity. Figure S3. The association of fruit-microbiota index with fruit categories in the Guangzhou Nutrition and Health Study. Figure S4. Relationships between the fruit intake-associated gut microbiota alterations and T2D-related traits. Figure S5. Associations of the vegetable/total fruit and vegetable intake-associated gut microbiota alterations with corresponding dietary intake and T2D. Figure S6. Association of the fruit-associated gut microbiota index and fecal metabolites. Figure S7. Fruit intake-related gut microbiota, fecal metabolites and T2D. Figure S8. Pathways enrichment analysis of the identified fecal metabolites associated with the fruit-related gut microbiota. [file 12916_2020_1842_MOESM1_ESM.docx]
